# Construction and validation of a cuproptosis-related prognostic model for glioblastoma

**DOI:** 10.3389/fimmu.2023.1082974

**Published:** 2023-02-06

**Authors:** Bohong Zhang, Lin Xie, Jiahao Liu, Anmin Liu, Mingliang He

**Affiliations:** ^1^Department of Anesthesiology, the Seventh Affiliated Hospital, Sun Yat-Sen University, Shenzhen, China; ^2^Department of Neurosurgery, Sun Yat-Sen Memorial Hospital, Sun Yat-Sen University, Guangzhou, China

**Keywords:** cuproptosis, prognosis, tumor microenvironment, risk score model, glioblastoma

## Abstract

**Background:**

Cuproptosis, a newly reported type of programmed cell death, takes part in the regulation of tumor progression, treatment response, and prognosis. But the specific effect of cuproptosis-related genes (CRGs) on glioblastoma (GBM) is still unclear.

**Methods:**

The transcriptome data and corresponding clinical data of GBM samples were downloaded from the TCGA and GEO databases. R software and R packages were used to perform statistical analysis, consensus cluster analysis, survival analysis, Cox regression analysis, Lasso regression analysis, and tumor microenvironment analysis. The mRNA and protein expression levels of model-related genes were detected by RT-qPCR and Western blot assays, respectively.

**Results:**

The expression profile of CRGs in 209 GBM samples from two separate datasets was obtained. Two cuproptosis subtypes, CRGcluster A and CRGcluster B, were identified by consensus cluster analysis. There were apparent differences in prognosis, tumor microenvironment, and immune checkpoint expression levels between the two subtypes, and there were 79 prognostic differentially expressed genes (DEGs). According to the prognostic DEGs, two gene subtypes, geneCluster A and geneCluster B, were identified, and a prognostic risk score model was constructed and validated. This model consists of five prognostic DEGs, including PDIA4, DUSP6, PTPRN, PILRB, and CBLN1. Ultimately, to improve the applicability of the model, a nomogram was established. Patients with GBM in the low-risk cluster have a higher mutation burden and predict a longer OS than in the high-risk group. Moreover, the risk score was related to drug sensitivity and negatively correlated with the CSC index.

**Conclusion:**

We successfully constructed a cuproptosis-related prognostic model, which can independently predict the prognosis of GBM patients. These results further complement the understanding of cuproptosis and provide new theoretical support for developing a more effective treatment strategy.

## Introduction

According to the traditional histopathological characteristics, gliomas can be divided into WHO I-IV types, among which GBM is the most malignant and belongs to WHO IV. Despite standard treatment, including surgery and chemoradiotherapy, the prognosis of GBM is dismal (1, 2). It effectively improves the clinical prognosis of GBM by *via* exploring new prognostic models, identifying high-risk patients, and providing precise treatment.

Cuproptosis, a novel form of programmed cell death, was first reported by Tsvetkov et al. in the journal of Science in March 2022. Different from the known apoptosis, pyroptosis, and ferroptosis, studies have indicated that, in the process of cuproptosis, Cu^2+^ combines with the lipoylated components of the tricarboxylic acid cycle in the mitochondrial respiratory chain, resulting in the aggregation of lipoylated protein and down-regulation of iron-sulfur cluster protein, followed by proteotoxic stress as well as cell death (3). In addition, the researchers preliminarily identified 12 CRGs, such as CDKN2A, PDHB, GLS, LIPT1, FDX1, DLD, MTF1, ATP7B, LIAS, DLAT, PDHA1, and SLC31A1 (3). In the past several months, prognostic models based on cuproptosis have been published in many kinds of tumors, such as head and neck squamous cell carcinoma (4), triple-negative breast cancer (5), lung adenocarcinoma (6), renal clear cell carcinoma (7), melanoma (8), hepatocellular carcinoma (9), and low-grade glioma (10), which accurately predict prognosis, tumor immune microenvironment, and response to chemotherapy or immunotherapy. However, no cuproptosis-related prognostic model has been reported in GBM. To explore the significance of CRGs in predicting the prognosis of GBM, this research intends to develop and verify a risk score model according to CRGs by analyzing the transcriptome expression profile as well as clinical parameters downloaded from the public databases, and further construct a nomogram to elevate the applicability of this model. Moreover, we conducted analyses of the TME, immune checkpoints, TMB, and sensitivities of drugs or compounds. This study may provide a novel insight into the prognostic prediction and precise treatments and ultimately improve the prognosis of GBM patients.

## Materials and methods

### Data collection

**Figure 1:**
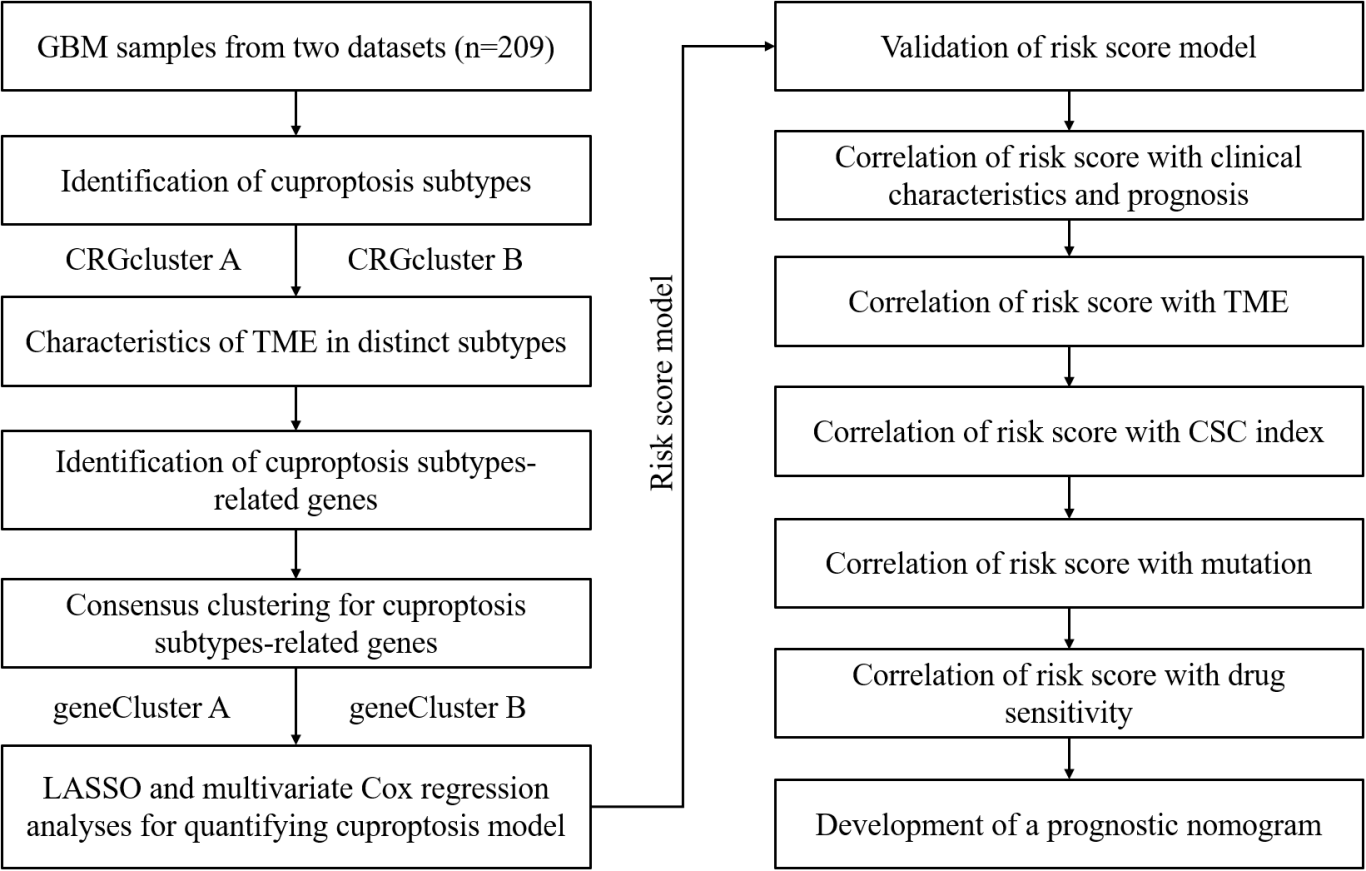
The entire analytical process of the study.

**Figure 1** displays the process of this research. The transcriptome expression profile (TPM) and associated clinical data for GBM were downloaded from public databases, including TCGA and GEO. We obtained a TCGA GBM cohort and two GEO GBM cohorts (GSE83300 cohort and GSE74187 cohort) for subsequent analyses. First, the original files were background adjusted and quantile normalized, and then the two datasets were combined after eliminating batch effects by applying the “Combat” algorithm. Excluding samples without OS information, 209 GBM samples were included. **Table S1** provides detailed clinical information about the 209 GBM patients, including age, sex, overall survival time, survival status, and IDH1 mutation status.

### Consensus clustering analysis of CRGs

Twelve CRGs were retrieved from previous publications. In consensus clustering analysis, the R package “ConsensusClusterPlus” was applied. The clustering criteria were as follows: the cumulative distribution function (CDF) curve is relatively flat rather than steep, the sample size of each subtype should not be too small, and the correlation within subtypes was enhanced after clustering, while the correlation between subtypes was weakened. Based on the expression profile of prognostic CRGs, samples were classified into distinct cuproptosis subtypes.

### Relationship of cuproptosis subtypes with clinical features

To identify the clinical significance of the classification above, analyses of the relationship between cuproptosis subtypes and age, sex, and prognosis were conducted, respectively. Using R packages “survminer” and “survival”, we conducted a Kaplan-Meier survival analysis to compare the difference of OS in different cuproptosis subtypes.

### Relationship between cuproptosis subtypes and TME

Based on the ssGSEA algorithm, we obtained the relative contents of 23 kinds of immune cells in every GBM sample. Furthermore, in light of the ESTIMATE algorithm, we obtained the ImmuneScore, StromalScore, and ESTIMATEScore of every GBM sample. Three of the most studied immune checkpoints were selected, including CTLA-4, PD-L1, and PD-1, and differentially expressed analyses of checkpoints between two cuproptosis subtypes were performed.

### DEG identification and consensus clustering analysis

Based on the R package “limma”, we obtained DEGs between the two cuproptosis subtypes with screening conditions of fold change (FC) > 1.5 and adjusted *P*< 0.05. Then, a univariate Cox regression analysis was conducted to obtain the prognostic DEGs. Finally, in light of the prognostic DEGs, unsupervised cluster analysis was performed to categorize GBM samples into distinct gene subtypes.

### Construct and validate the cuproptosis-related risk score model

GBM samples were randomly categorized into the training set and the testing set. Sample sizes of the two sets were about the same, with 105 GBM patients in the training set and 104 GBM patients in the testing set. We chose the training set to establish a risk score model. Briefly, using the R package “glmnet”, Lasso Cox regression analysis was conducted in light of the prognostic DEGs. We established the risk score model by analyzing the change trajectory of every gene and using the 10-fold cross-validation method.

The calculation formula is as follows: *Risk* *score* = *Σ* (*Coefi* × *Exp*) In this formula, Coefi indicates the risk coefficient, and Exp indicates the expression level. In the training set, samples were categorized into high-risk and low-risk clusters in light of the median. Then a Kaplan-Meier survival analysis was conducted to compare the OS difference between the high-risk and low-risk clusters. Next, using the R package “ggplot2”, PCA was performed to observe the distinction between the two risk clusters. Finally, the ROC curve was constructed to assess the prognostic prediction performance of this model. For further verification, samples in the testing set, GSE83300 cohort, and GSE74187 cohort were categorized based on the median risk score, respectively, followed by Kaplan-Meier survival analysis, PCA, and ROC curve construction.

### Tissue samples collection

The Ethics Committee permitted this experiment, and all included patients signed informed consent. Five pairs of GBM specimens and nearby non-tumor specimens were collected from the Department of Neurosurgery from 2017 to 2020. Samples were stored at -80°C immediately after resection and then transferred to liquid nitrogen.

### RNA isolation and qRT-PCR

Firstly, based on the manufacturer’s instructions, tissue RNA was extracted by Trizol (Invitrogen, USA). Secondly, reverse transcription was conducted by PrimeScript™ RT Mix (Takara) containing random and oligo primers to obtain cDNA. Finally, qPCR analysis was performed on a Light-Cycler 480 qPCR instrument with LightCycler^®^ 480 SYBR Master (Roche). Here are the PCR conditions: preincubation at 96°C for 6 minutes, 39 cycles of amplification with 9 s at 93°C, 19 s at 58°C, followed by an extension at 68°C for 16 s. The expression levels of target genes were obtained by a formula of 2^-ΔΔCt^. **Table S2** provides sequences of primers used in this research.

### Western blotting

The protein expression levels of model-related genes were verified by Western blotting. Samples were treated with RIPA lysis buffer. A BCA assay kit (Thermofisher, USA) was used to determine the protein concentration. An equal amount of protein (20 μg) was loaded into lanes. After being separated by electrophoresis, proteins were electrically transferred to a PVDF membrane (Millipore, USA). After blockaded with 5% milk, the membrane was incubated with the primary antibodies: anti-PDIA4 (SAB1404743, Sigma-Aldrich, St. Louis, MO, USA), anti-DUSP6 (SAB1410312, Sigma-Aldrich, St. Louis, MO, USA), anti-PTPRN (ab207750, Abcam, Cambridge, MA, USA), anti-PILRB (ab198267, Abcam, Cambridge, MA, USA), anti-CBLN1 (ab181379, Abcam, Cambridge, MA, USA), and anti-GAPDH (ab181602, Abcam, Cambridge, MA, USA), each was diluted at a ratio of 1:1000 and incubated over-night at 4 °C. After incubation with the corresponding horseradish peroxidase-linked secondary antibody at room temperature for 1 h, target proteins were developed by the enhanced chemiluminescence kit (Millipore, USA).

### Relationship analyses between clinical characteristics and risk score and stratification analyses

Three clinical characteristics, age, gender, and IDH1 mutation status, were selected, and samples were categorized based on each clinical characteristic. Then we conducted a differential analysis of risk score with the Chi-square test. We conducted univariate and multivariate regression analyses to verify if the risk score is an independent prognostic factor. Moreover, we conducted stratification analyses. Briefly, the samples were stratified according to each selected clinical characteristic. The stratified samples were further stratified in light of the median risk score, followed by Kaplan-Meier survival analyses to observe if the OS difference in each subgroup was statistically significant.

### Relationship of risk score with immune status and cancer stem cell index

In light of the CIBERSORT algorithm, we obtained the relative contents of 22 types of immune cells in every GBM sample. Next, we conducted correlation analyses between risk scores and the contents of immune cells and the correlation analyses between the contents of immune cells and the expression of model-related genes. Moreover, in light of the ESTIMATE algorithm, we obtained the ImmuneScore, StromalScore, and ESTIMATEScore of every GBM sample. The differential analyses of ImmuneScore, StromalScore, and ESTIMATEScore between the high-risk and low-risk clusters were conducted. Moreover, we picked multiple immune checkpoint molecules followed by differential analyses of immune checkpoint expression levels between the two risk clusters. Finally, a correlation analysis between CSC indexes and risk scores was conducted. The CSC index is an indicator describing the degree of similarity between tumor cells and stem cells, which can be considered as the quantification of CSCs. Each CSC index ranges from low (zero) to high (one) stemness. The CSC index was calculated using the innovative one-class logistic regression (OCLR) machine learning algorithm (11).

### Mutation analyses and drug sensitivity analyses

Based on the R package “maftools”, we obtained the TMB of every GBM sample. Next, the differential analysis of TMB between the two risk clusters was conducted. Moreover, we calculated the IC50 values of chemotherapeutic drugs commonly used using the R package “pRRophetic”. It’s an R package predicting chemotherapeutic response based on tumor gene expression profiles (12). Next, differential analyses of the IC50 values between the high-risk and low-risk clusters were performed to assess the differences in therapeutic effects.

### Construct a nomogram to improve the applicability

Using the R package “rms”, a predictive nomogram was constructed according to clinical features and risk score. A patient’s score equals the sum of the corresponding scores for every variable. Based on the total score, we can predict the survival rates of a patient at 0.5, 1.0, and 1.5 years. Moreover, ROC curves were constructed to obtain the AUC values of 0.5, 1.0, and 1.5 years to assess the performance of this nomogram. Finally, a calibration plot was established to observe the differences between the predicted survival events of 0.5, 1.0, and 1.5 years and the actual results, thus further assessing the performance of this nomogram.

### Statistical analyses

R was used for most of the statistical analyses in this study.

## Results

### Differential expression of CRGs between the tumor and normal tissues

**Figure 2:**
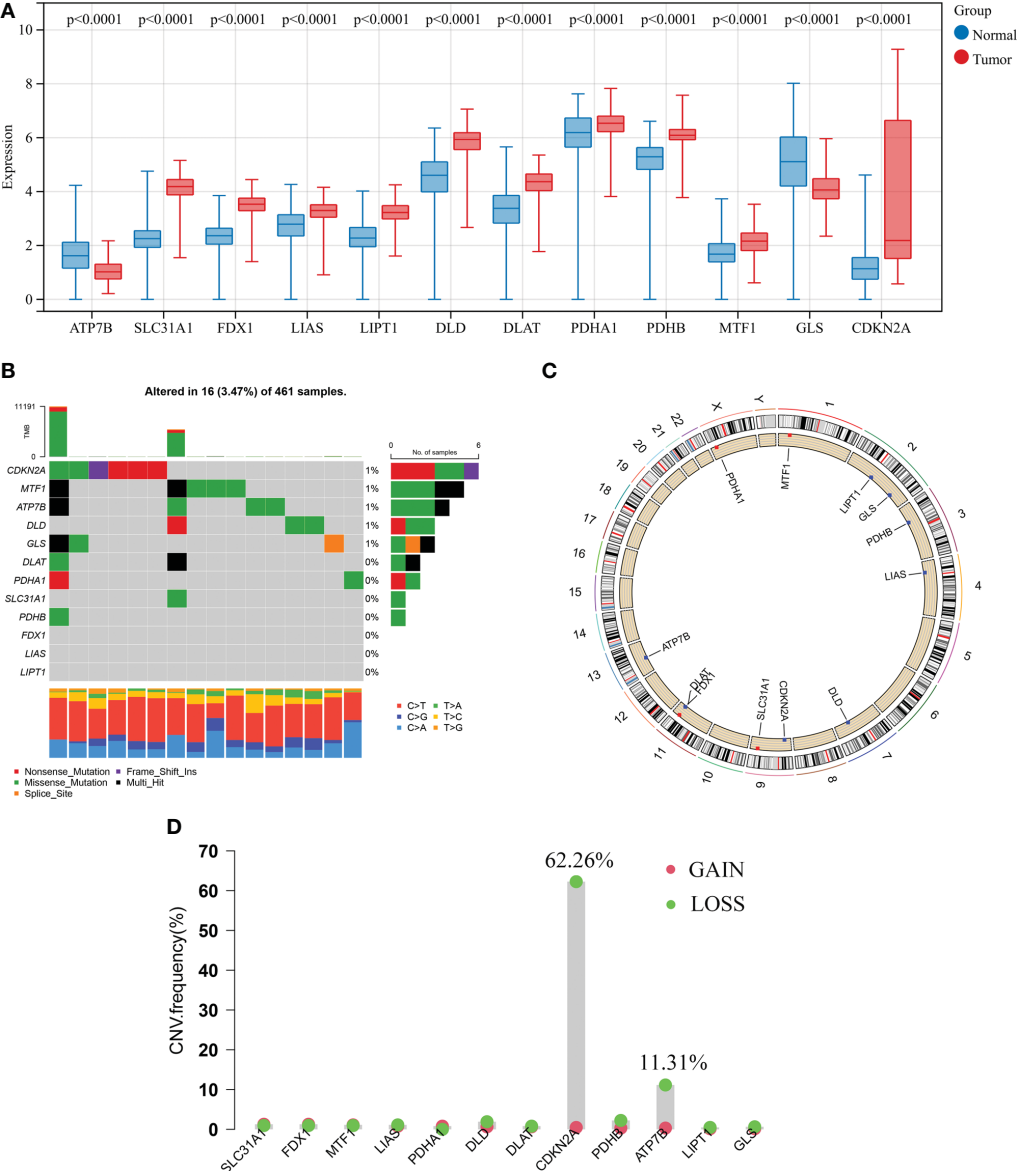
Transcriptional and genetic alterations of 12 CRGs in GBM. **(A)** Differentially expressed analyses of 12 CRGs between normal and GBM samples. **(B)** Mutation frequencies of 12 CRGs in 461 patients with GBM from the TCGA cohort. **(C)** Locations of CNV alterations in CRGs on 23 chromosomes. **(D)** Frequencies of CNV gain, loss, and non-CNV among CRGs. CRGs, cuproptosis-related genes; GBM, glioblastoma; TCGA, The Cancer Genome Atlas; CNV, copy number variant.

In this study, a total of 12 CRGs were included. We conducted differentially expressed analyses of CRGs between normal and GBM samples and found that 10 CRGs were significantly up-regulated in GBM samples, including SLC31A1, CDKN2A, MTF1, LIPT1, FDX1, PDHB, PDHA1, LIAS, DLD, and DLAT. Conversely, 2 CRGs were significantly down-regulated, including ATP7B and GLS (**Figure 2A**).

Next, analysis of somatic mutations in 12 CRGs revealed a low-frequency mutation in GBM samples. Of 461 GBM samples, there were 16 (3.47%) mutations in CRGs. Five CRGs (*CDKN2A*, *MTF1*, *ATP7B*, *DLD*, and *GLS*) had a mutation frequency of 1%, respectively, while three CRGs (*FDX1*, *LIAS*, and *LIPT1*) did not have any mutations (**Figure 2B**). Furthermore, we analyzed the copy number variation (CNV) in 12 CRGs and discovered frequent CNV in only 2 CRGs. CDKN2A and ATP7B showed an apparent CNV decrease (**Figure 2D**). **Figure 2C** displays the locations of 12 CNV on their respective chromosomes. CRG with CNV loss, such as ATP7B, the expression level was lower in the GBM sample than that in the normal sample, while the other CRG with CNV loss, CDKN2A, was significantly elevated in the GBM sample. Besides, other significantly differentially expressed CRGs showed very low frequencies of CNV.

### Identification of cuproptosis subtypes in GBM

**Figure 3:**
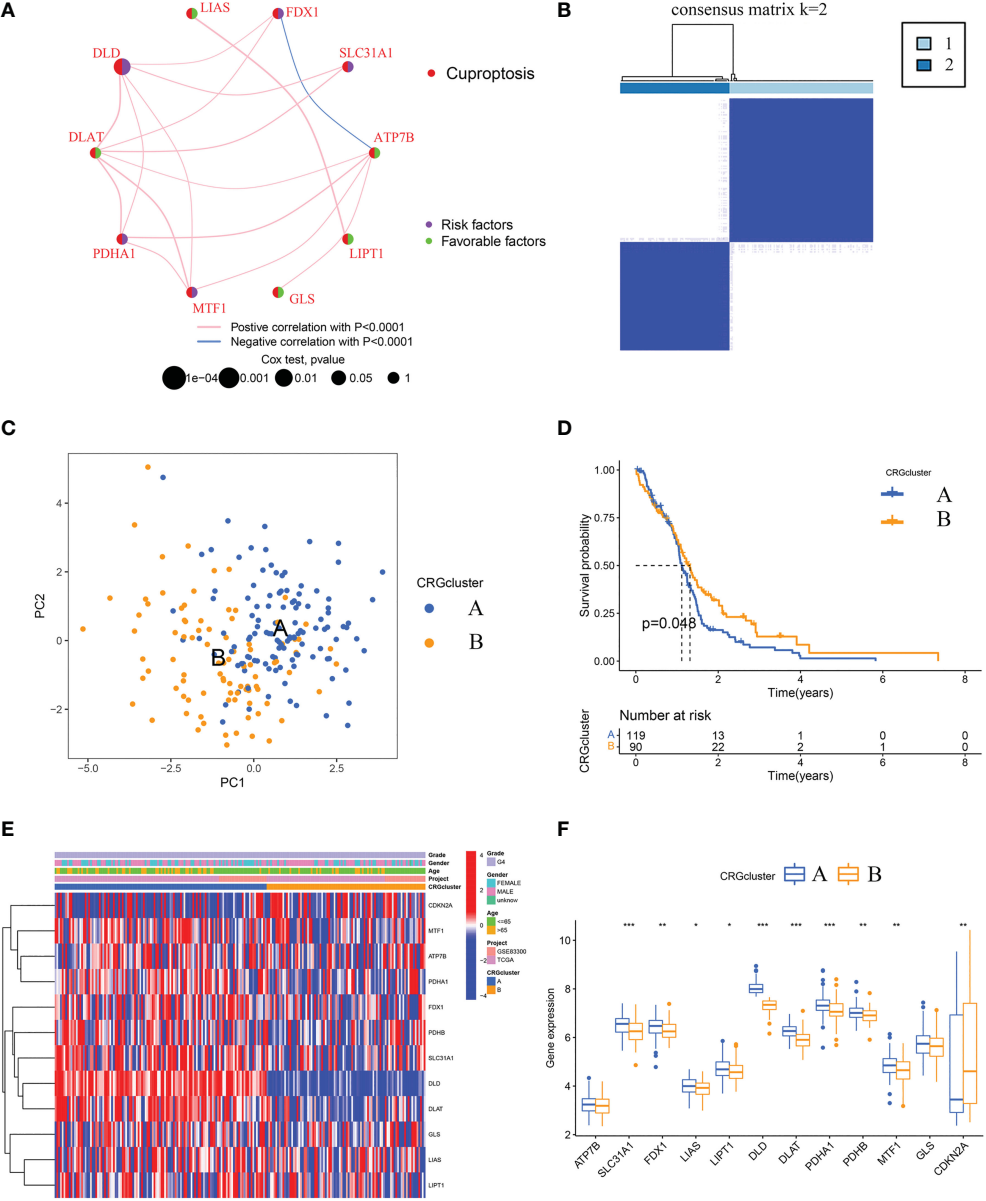
Identification of cuproptosis subtypes and comparison of clinical characteristics and CRGs expression levels between the two subtypes. **(A)** Interaction among CRGs in GBM. The line connecting the CRGs represents their interaction, with the line thickness indicating the strength of the association between CRGs. Pink lines and blue lines represent positive and negative correlations, respectively. **(B)** Unsupervised clustering analysis of prognostic CRGs. Consensus matrix heatmap defining two clusters (k = 2) and their correlation area. **(C)** The PCA analysis based on the prognostic CRGs demonstrated that the patients in the different cuproptosis subtypes were distributed in two directions. Blue and yellow dots represent CRGcluster A and CRGcluster B. **(D)** Kaplan-Meier curves for OS of the two cuproptosis subtypes (chi-square test, *p* = 0.048). **(E)** The heat map shows the differences in clinical features and CRGs expression levels between CRGcluster A and CRGcluster **(B, F)** Box plot shows the differences of CRGs expression levels between CRGcluster A and CRGcluster B. CRGs, cuproptosis-related genes; GBM, glioblastoma; PCA, principal components analysis; OS, overall survival. *p< 0.05, **p< 0.01, ***p< 0.001 *vs.* CRGcluster A.

A total of 209 patients from two GBM cohorts (TCGA-GBM and GSE83300 cohort) were merged. Detailed information on the 209 GBM samples was obtained. The prognostic significance of 12 CRGs was determined by Kaplan-Meier survival analyses and univariate Cox regression analyses. Five CRGs, including ATP7B, CDKN2A, DLD, MTF1, and SLC31A1, were identified as prognostic CRGs (**Table S3**). As shown in **Figure 3A**, a cuproptosis network systematically revealed the interactions and prognostic significances of CRGs in GBM.

Further, according to the expression profiles of the five prognostic CRGs, GBM samples were classified by consensus clustering algorithm. The consensus matrix heatmap showed that k = 2 is the optimal classification method, and the GBM samples were classified into CRGcluster A (number of samples = 119) and CRGcluster B (number of samples = 90) (**Figure 3B**). PCA indicated that the cuproptosis transcription profiles of the two subtypes were different (**Figure 3C**). Moreover, Kaplan-Meier curves showed that GBM samples in CRGcluster B had a longer OS than samples in CRGcluster A (chi-square test, *P* = 0.048; **Figure 3D**). By comparing the clinical characteristics of the two subtypes, no noticeable difference in age and sex was observed (**Figure 3E**). While the expression levels of most CRGs in CRGcluster A were higher than that in CRGcluster B (**Figures 3E, F**).

### Characteristics of the TME in distinct cuproptosis subtypes

**Figure 4:**
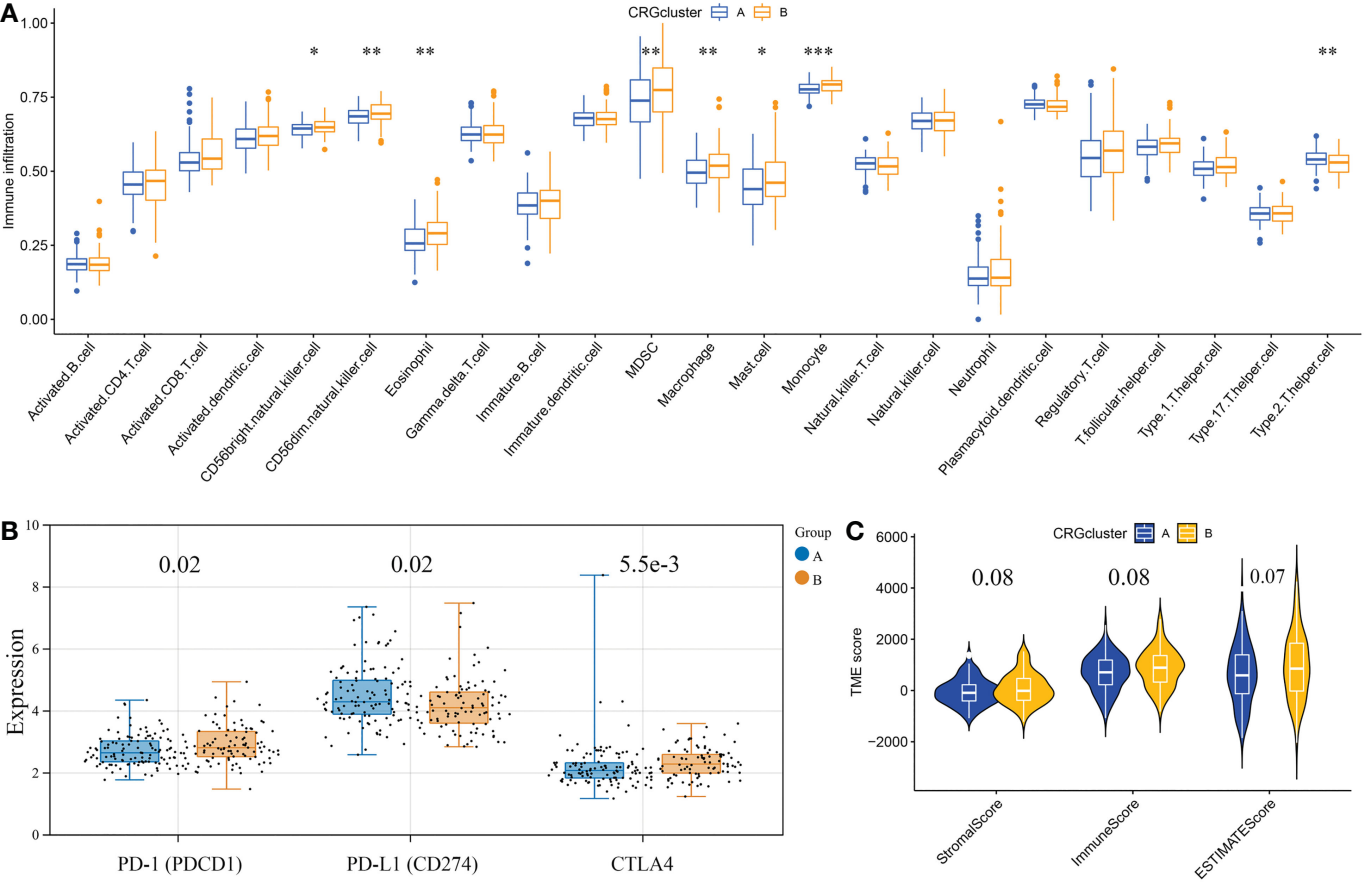
Correlations of the tumor microenvironment and the two cuproptosis subtypes. **(A)** The abundance of 23 infiltrating immune cell types in the two cuproptosis subtypes. **(B)** The expression levels of PD-1, PD-L1, and CTLA4 in the two cuproptosis subtypes. The blue boxes represent CRGcluster A, and the yellow boxes represent CRGcluster B. **(C)** The violin diagram shows the three kinds of TME scores in CRGcluster A and CRGcluster B. *p< 0.05, **p< 0.01, ***p< 0.001 *vs.* CRGcluster A.

First, based on the ssGSEA algorithm, we obtained the relative contents of 23 kinds of immune cells in every GBM sample. And the relative contents of some kinds of immune cells, including MDSCs, CD56^+^ natural killer cells, macrophages, eosinophils, type 2 T helper cells, mast cells, monocytes, and CD56^-^ natural killer cells, significantly differed between the two subtypes (**Figure 4A**). As for the immune checkpoints, the expression level of PD-L1 in CRGcluster B was lower than that in CRGcluster A, while the expression levels of CTLA4 and PD-1 were higher than that in CRGcluster A (**Figure 4B**). Moreover, using the ESTIMATE algorithm, we obtained the TME scores of every GBM sample, including ImmuneScore, StromalScore, and ESTIMATEScore. ImmuneScore represents the content of the immune component, StromalScore represents the content of the matrix component, and ESTIMATEScore is the sum of the two. The differential analysis showed that TME scores were slightly higher in CRGcluster B, but no significant difference was observed (**Figure 4C**).

### Identification of prognostic DEGs and classification of gene subtypes

**Figure 5:**
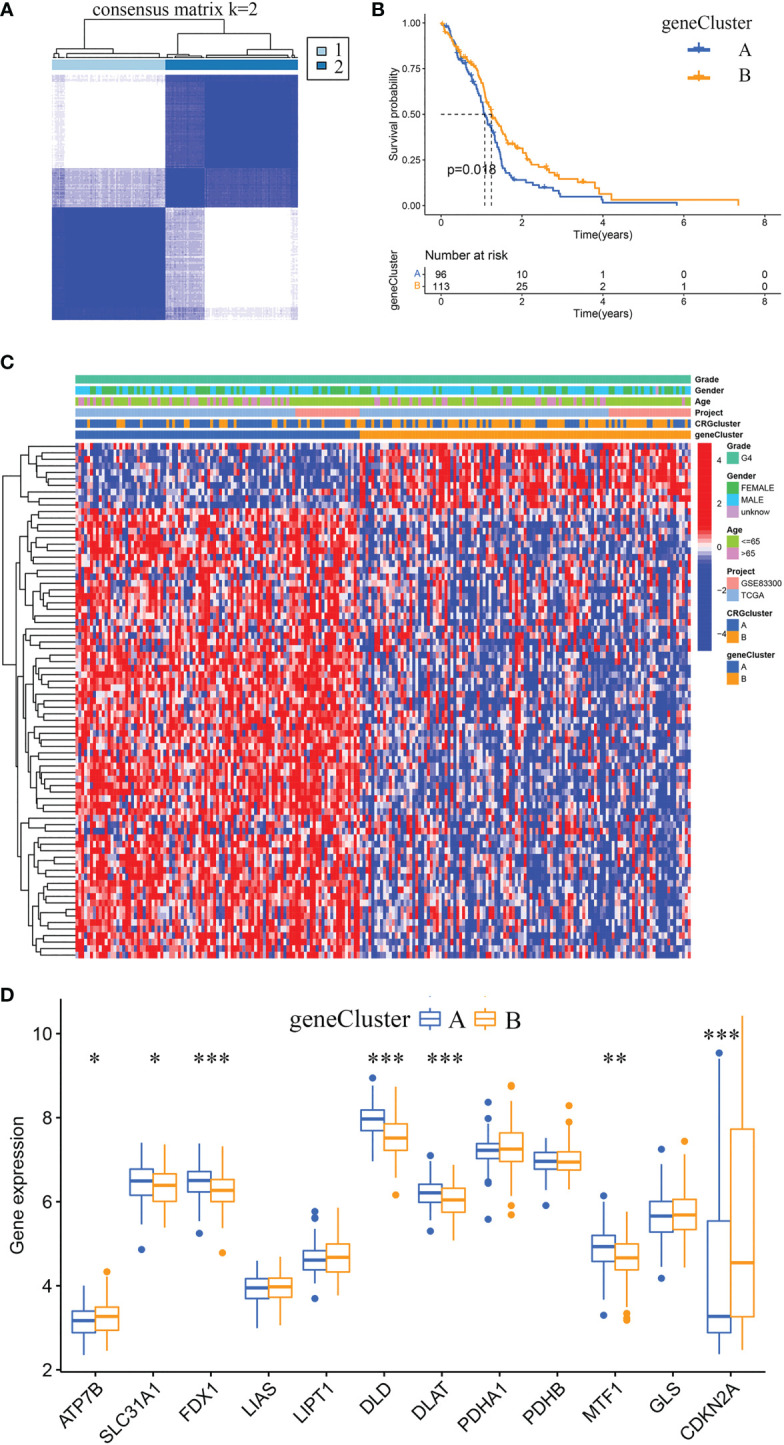
Identification of gene subtypes and comparison of clinical characteristics and CRGs expression levels between the two gene subtypes. **(A)** Unsupervised clustering analysis of prognostic DEGs between the two cuproptosis subtypes. Consensus matrix heatmap defining two clusters (k = 2) and their correlation area. **(B)** Kaplan-Meier curves for OS of the two gene subtypes (chi-square test, *p* = 0.018). **(C)** The heat map shows the differences in clinical features, and prognostic DEGs expression levels between geneCluster A and geneCluster B. **(D)** Box plot shows the differences of CRGs expression levels between geneCluster A and geneCluster B. DEGs, differentially expressed genes; CRGs, cuproptosis-related genes. *p< 0.05, **p< 0.01, ***p< 0.001 *vs.* geneCluster A.

Using the R package “limma”, we identified 360 cuproptosis subtype-related DEGs. And *via* univariate Cox regression analysis, we identified 79 prognostic DEGs. Furthermore, according to the expression profiles of the 79 prognostic DEGs, GBM samples were classified by consensus clustering algorithm. The consensus matrix heatmap showed that k = 2 is the optimal classification method, and the GBM samples were classified into geneCluster A (number of samples = 96) and geneCluster B (number of samples = 113) (**Figure 5A**). Kaplan-Meier curves revealed that GBM samples in geneCluster B had a longer OS than in geneCluster A (chi-square test, *P* = 0.018; **Figure 5B**). By comparing the clinical characteristics of the two subtypes, no noticeable difference in age and sex was observed. While the expression levels of most prognostic DEGs in geneCluster A were higher than in geneCluster B (**Figure 5C**). The differentially expressed analysis of 12 CRGs between geneCluster A and geneCluster B is shown in **Figure 5D**.

### Established and validated a risk score model

**Figure 6:**
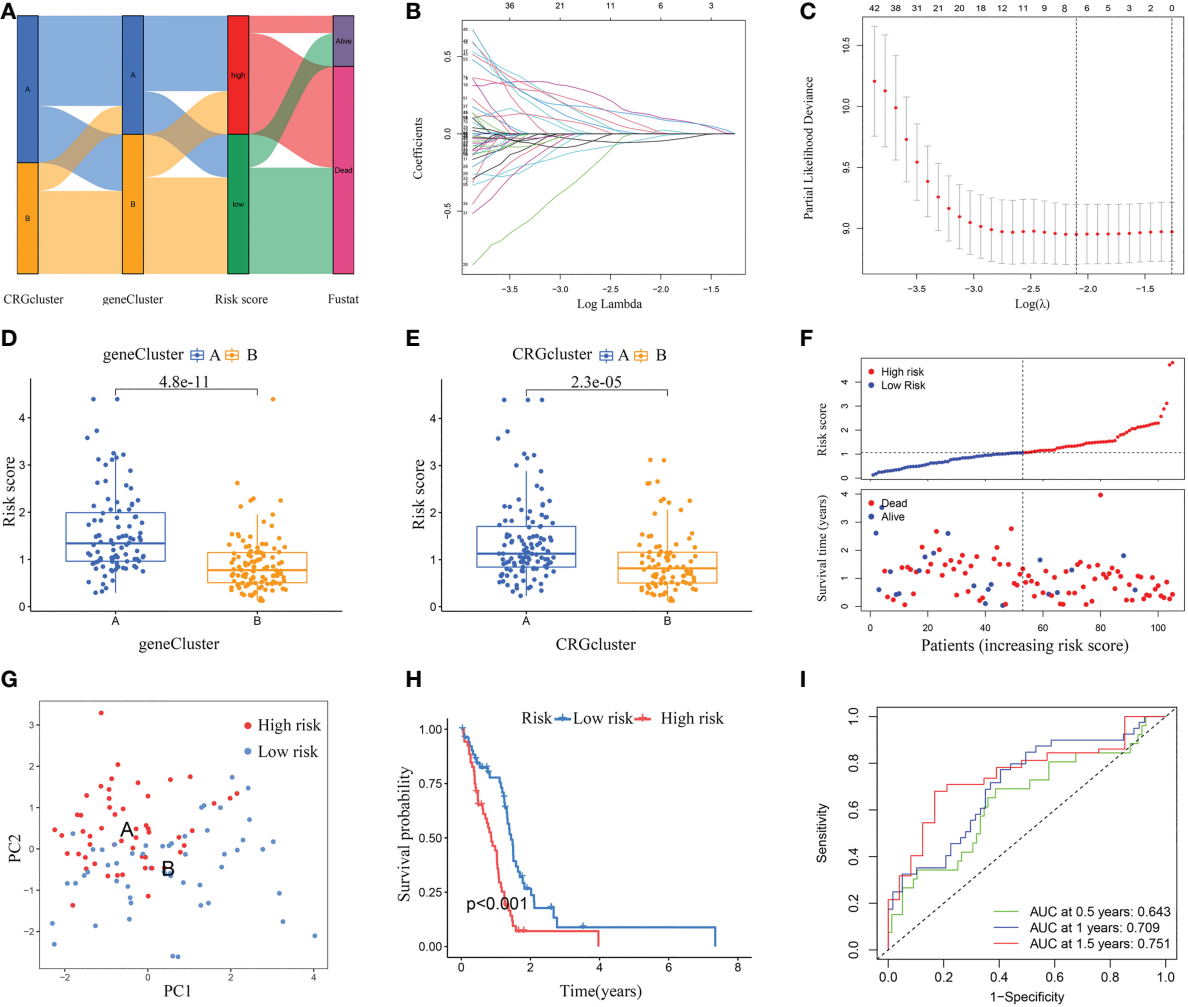
Constructed a risk score model in the training set. **(A)** Alluvial diagram of subtype distributions and prognosis of GBM patients. **(B, C)** LASSO regression analysis identified representative candidate prognostic genes and partial likelihood deviance on the prognostic genes. **(D)** The difference in risk scores between the two gene subtypes. **(E)** The difference in risk scores between the two cuproptosis subtypes. **(F)** The ranked dot plot indicates the risk score distribution, and the scatter plot presents the patients’ survival status. **(G)** The PCA analysis based on the prognostic signature demonstrated that the patients in the different risk score groups were distributed in two directions. Red and blue dots represent the high-risk group and the low-risk group. **(H)** Kaplan-Meier curves for OS of the two risk groups (chi-square test, *p*< 0.001). **(I)** ROC curves to predict the sensitivity and specificity of 0.5-, 1.0-, and 1.5-year survival according to the risk score. ROC, receiver operating characteristic.

According to cuproptosis subtype-related DEGs, a prognostic risk score model was developed. **Figure 6A** displays the distribution of GBM samples by different classification methods. Firstly, patients with GBM were randomly categorized into training and testing sets. Sample sizes of the two sets were about the same, with 105 GBM samples in the training set and 104 GBM samples in the testing set. Secondly, we conducted LASSO regression analysis of the 79 prognostic DEGs and obtained seven candidate genes for the risk score model (**Figures 6B, C**), followed by multivariate Cox analysis of the seven candidate genes, and finally obtained five target genes (*PDIA4*, *PILRB*, *DUSP6*, *CBLN1*, and *PTPRN*), including four high-risk genes (*PDIA4*, *PILRB*, *DUSP6*, and *PTPRN*) and one low-risk gene (*CBLN1*). Calculation formula: Risk score = (0.2988 × *PDIA4*) + (0.1705 × *PILRB*) + (0.2448 × *DUSP6*) + (0.3055 × *PTPRN*) + (-0.2095 × *CBLN1*).

The differential analyses indicated that the risk scores of geneCluster A and CRGcluster A were higher than that of geneCluster B and CRGcluster B, respectively (**Figures 6D, E**). In the training set, according to the median, GBM samples were classified into a low-risk cluster (n = 53) and a high-risk cluster (n = 52). With the increase in risk score, the OS of GBM patients gradually decreased, and the deaths steadily increased (**Figure 6F**). PCA clearly distinguishes the two risk clusters (**Figure 6G**). Survival curves revealed that GBM samples in the low-risk cluster had a longer OS than in the high-risk group (chi-square test, *p*< 0.001; **Figure 6H**). Furthermore, AUC values of 0.5-, 1.0-, and 1.5-year based on this model were 0.643, 0.709, and 0.751, respectively (**Figure 6I**).

**Figure 7:**
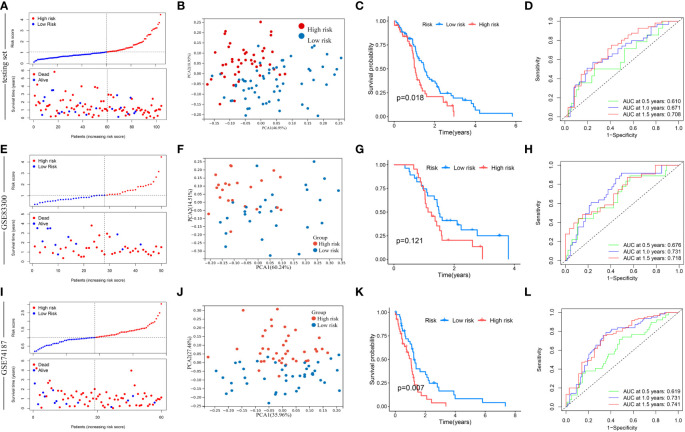
Validation of risk score in the testing set, GSE83300 cohort, and GSE74187 cohort. **(A, E, I)** The ranked dot plot indicates the risk score distribution, and the scatter plot presents the patients’ survival status. **(B, F, J)** The PCA analysis based on the prognostic signature demonstrated that the patients in the different risk score groups were distributed in two directions. Red and blue dots represent the high-risk group and the low-risk group. **(C)** Kaplan-Meier curves for OS of the two risk groups (chi-square test, *p* = 0.018). **(G)** Kaplan-Meier curves for OS of the two risk groups (chi-square test, *p* = 0.121). **(K)** Kaplan-Meier curves for OS of the two risk groups (chi-square test, *p* = 0.007). **(D, H, L)** ROC curves to predict the sensitivity and specificity of 0.5-, 1.0-, and 1.5-year survival according to the risk score.

Next, this model was verified in the testing set, GSE83300 cohort, and GSE74187 cohort, respectively (**Figure 7**). According to the median risk score, GBM samples of the testing set, GSE83300 cohort, and GSE74187 cohort were categorized, respectively. With the increase in risk score, the OS of GBM patients gradually decreased, and the deaths steadily increased (**Figures 7A, E, I**). PCA clearly distinguishes the two risk clusters (**Figures 7B, F, J**). In the testing set, Kaplan-Meier curves revealed that GBM in the low-risk cluster had a longer OS than in the high-risk group (chi-square test, *p* = 0.018; **Figure 7C**). In the GSE83300 cohort, although the result of survival analysis did not reach statistical significance, GBM samples in the low-risk cluster tended to prolonged OS (chi-square test, *p* = 0.121; **Figure 7G**). In the GSE74187 cohort, Kaplan-Meier curves revealed that GBM in the low-risk cluster had a longer OS than in the high-risk group (chi-square test, *p* = 0.007; **Figure 7K**). In the testing set, AUC values of 0.5-, 1.0-, and 1.5-year survival rates based on this model were 0.610, 0.671, and 0.708, respectively (**Figure 7D**). In the GSE83300 cohort, the AUC values of 0.5-, 1.0-, and 1.5-year survival rates based on this model were 0.676, 0.731, and 0.718, respectively (**Figure 7H**). In the GSE74187 cohort, the AUC values of 0.5-, 1.0-, and 1.5-year survival rates based on this model were 0.619, 0.731, and 0.741, respectively (**Figure 7l**).

### Validated the expression of the five model-related genes

**Figure 8:**
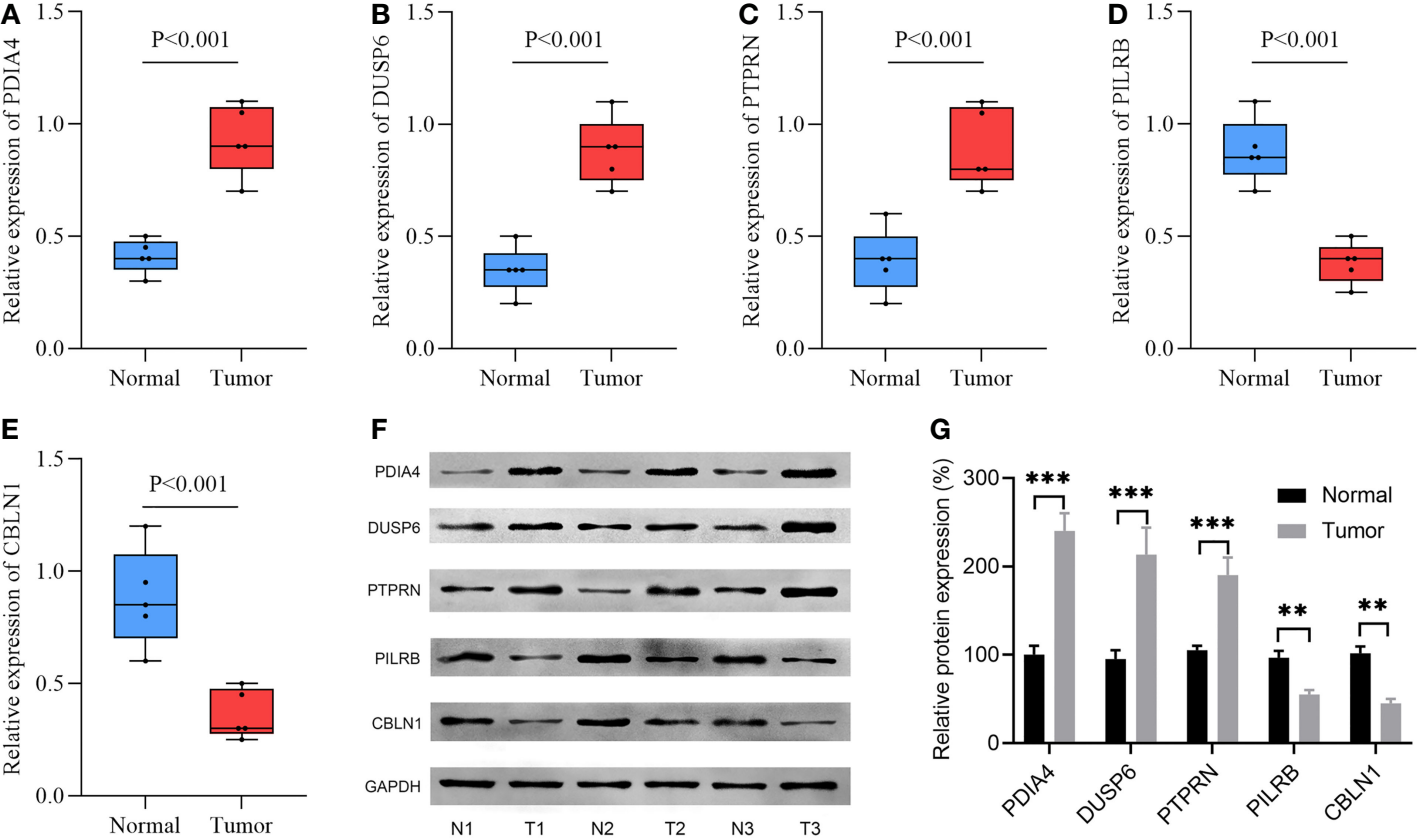
The expression levels of 5 model-related genes in GBM tissues and corresponding normal tissues were validated by RT-qPCR and WB. **(A–E)** RT-qPCR validated the mRNA expression levels of genes. **(F)** WB assay validated the protein expression levels of genes. **(G)** Relative density is determined by the densitometry of the blots. N, normal; T, tumor. **p< 0.01, ***p< 0.001 *vs.* normal group.

Using RT-qPCR assay, the mRNA expression levels of the five model-related genes in five pairs of GBM and adjacent tissues were detected. Compared with the paired adjacent specimens, the expression levels of PDIA4, DUSP6, and PTPRN were upregulated; however, the expression levels of PILRB and CBLN1 were downregulated in GBM specimens (**Figures 8A–E**). To validate the protein expression levels of the five model-related genes, a WB assay was conducted. Consistently, compared with the paired adjacent specimens, the protein expression levels of PDIA4, DUSP6, and PTPRN were upregulated, and the protein expression levels of PILRB and CBLN1 were downregulated in GBM specimens (**Figures 8F, G**).

### Clinical correlation analysis and stratification analysis of the prognostic risk score

**Figure 9:**
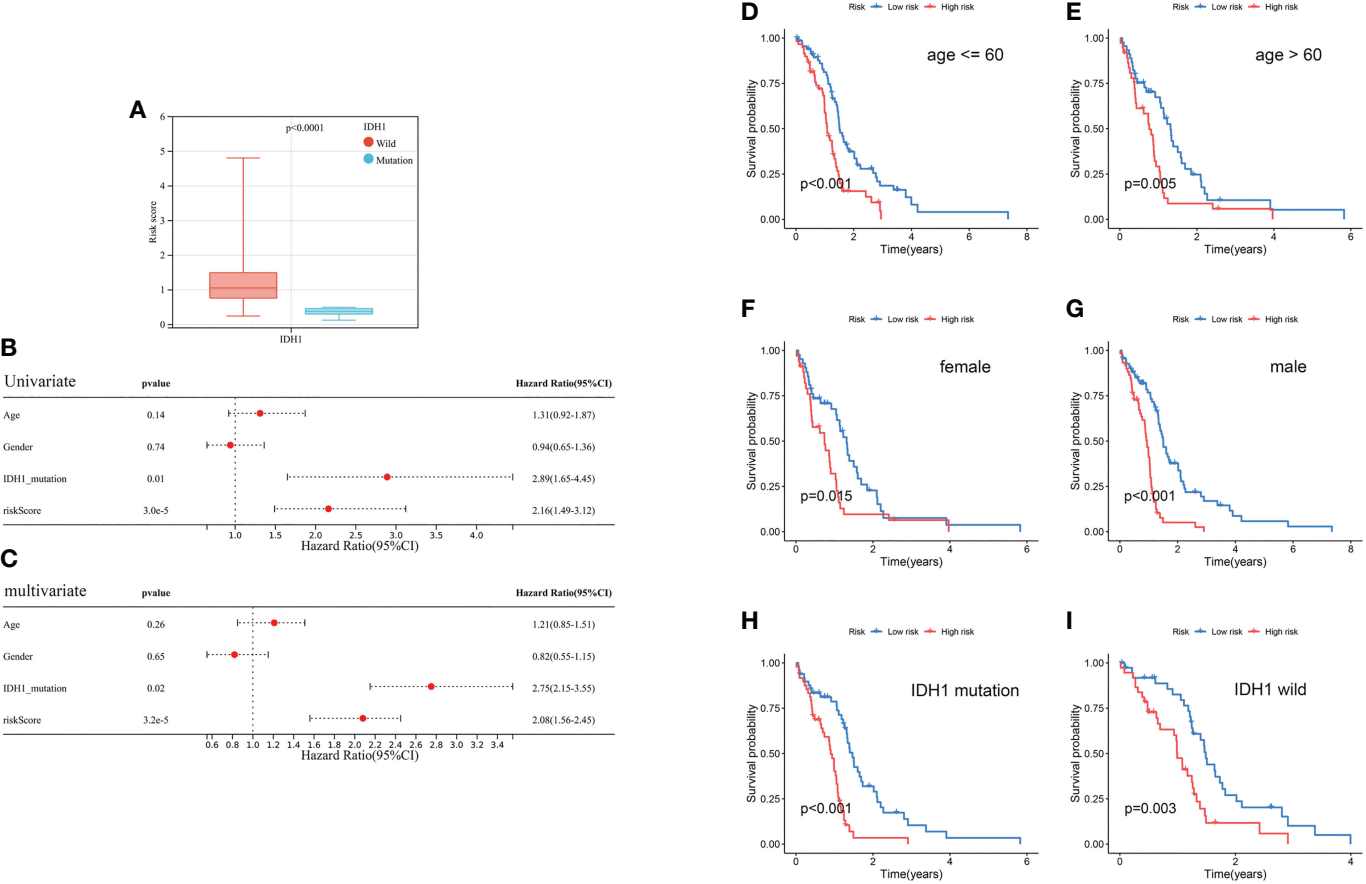
Clinical correlation analysis and stratification analysis of the prognostic risk score. **(A)** The correlation between the risk score and IDH1 mutation status. **(B, C)** Univariate and multivariate analyses showed the prognostic value of the risk score and IDH1 mutation. **(D, E)** Stratification analysis of the risk score in GBM. Age (age ≤ 60 and age > 60 years old). **(F, G)** Gender (female and male). **(H, I)** IDH1 mutation status (mutant type and wild type).

The risk score in the IDH1 mutant cluster was lower than in the IDH1 wild-type cluster (*P*< 0.0001; **Figure 9A**). Results of univariate and multivariate regression analyses revealed that both prognostic risk score and IDH1 mutation status were independent prognostic factors (**Figures 9B, C**). In addition, stratification analyses were performed to evaluate if the model had broad applicability. Kaplan-Meier curves revealed that GBM in the low-risk cluster always had a longer OS than in the high-risk group, and *p*< 0.001 in the subcluster of age younger than 60, *p* = 0.005 in the subcluster of age older than 60, *p<* 0.001 in subcluster of male, *p* = 0.015 in subcluster of female, *p*< 0.001 in subcluster of IDH1 mutation, and *p* = 0.003 in subcluster of IDH1 wild-type (**Figures 9D–I**).

### Evaluation of TME and checkpoints between the two risk score groups

**Figure 10:**
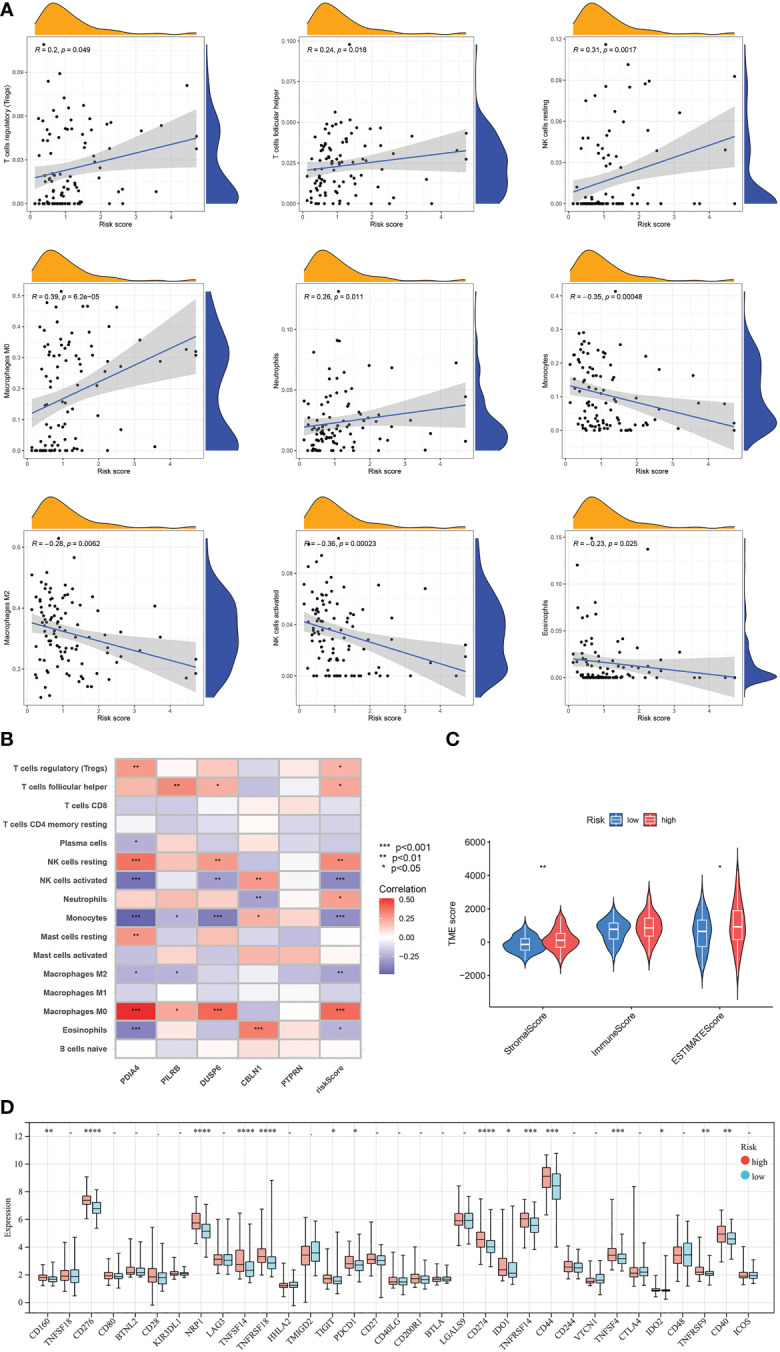
Evaluation of the TME and checkpoints between the two risk score groups. **(A)** Correlation analyses between risk scores and the contents of immune cells. **(B)** Correlation heatmap of Spearman correlation analyses between 5 model-related genes and the contents of immune cells. **(C)** The violin diagram shows the three kinds of TME scores in the high-risk group and low-risk group. **(D)** Differentially expressed analyses of immune checkpoints between the high-risk group and low-risk group. *p < 0.05, **p < 0.01, ***p < 0.001, ****p < 0.0001, –*p* ≥ 0.05 vs. low-risk group.

Results of correlation analyses between the risk scores and the contents of immune cells indicated that they were positively correlated in regulatory T cells, follicular helper T cells, neutrophils, resting NK cells, and M0 macrophages, while negatively correlated in eosinophils, M2 macrophages, monocytes, and activated NK cells, (**Figure 10A**). Moreover, correlation analyses between the contents of immune cells and the expression levels of 5 model-related genes showed obvious correlations between some kinds of immune cells and specific genes (**Figure 10B**). Furthermore, GBM samples in the low-risk cluster had a significantly lower StromalScore and ESTIMATEScore than samples in the high-risk group (**Figure 10C**). Finally, differentially expressed analyses of immune checkpoints between the high-risk cluster and the low-risk cluster were conducted. Results revealed that the expression levels of 15 immune checkpoints were lower in the low-risk cluster than in the high-risk cluster, for example, PD-1 and PD-L1 (**Figure 10D**).

### Evaluated the association of risk score with CSC index, TMB, and drug sensitivity

**Figure 11:**
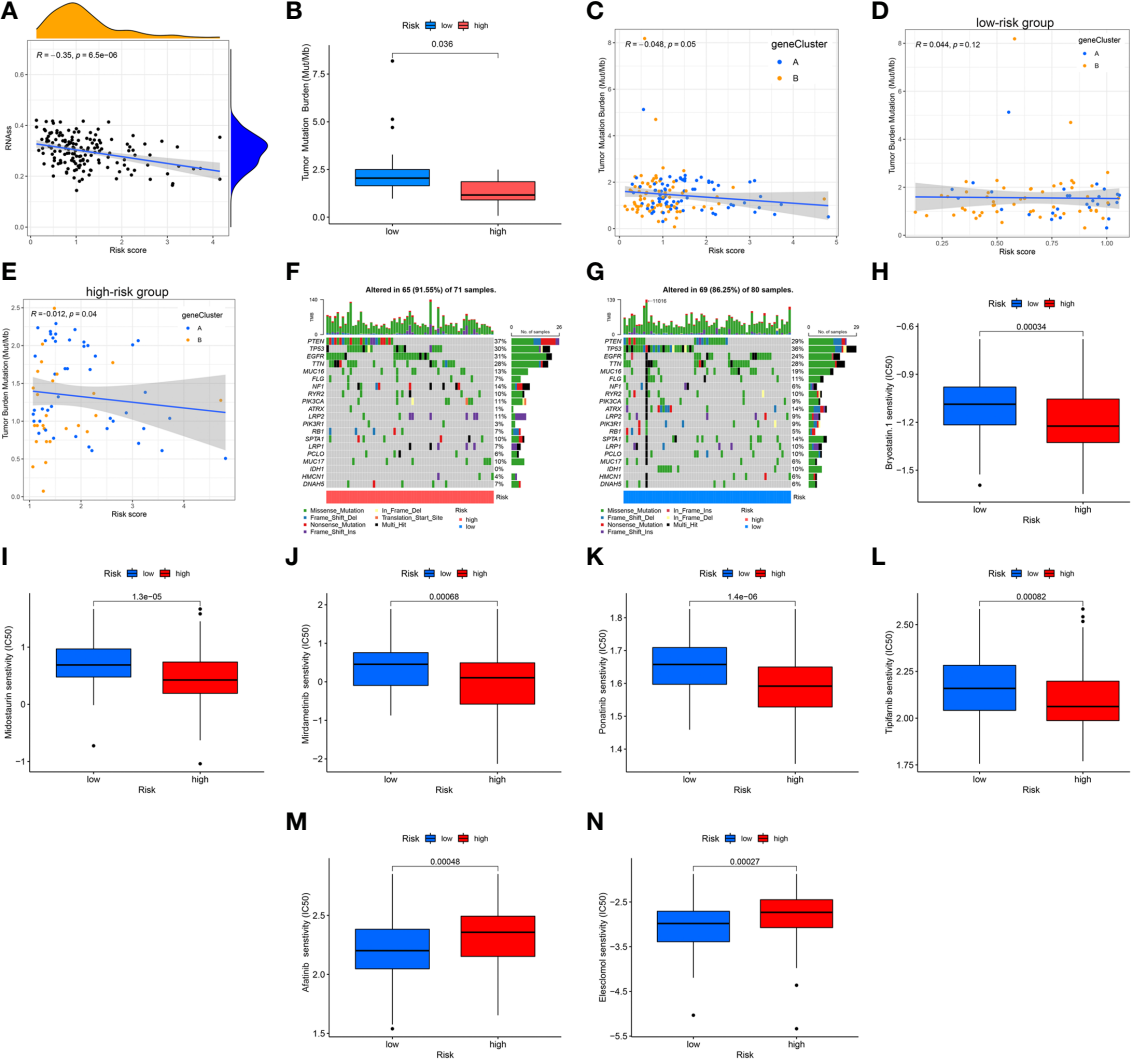
Association of risk score with CSC index, TMB, and drug sensitivity. **(A)** Correlation analysis between CSC indices and risk scores. **(B)** Differential analysis of TMB between the two risk groups. **(C–E)** Spearman correlation analysis between TMB and risk scores. **(F, G)** The waterfall plots of somatic mutation feature established with high- and low-risk scores. Each column represented an individual patient. The upper barplot showed TMB, and the number on the right indicated the mutation frequency in each gene. The right barplot showed the proportion of each variant type. **(H–N)** Differential analyses of IC50 values of common drugs or compounds. CSC, cancer stem cell; TMB, tumor mutation burden.

Results of the correlation analysis between the CSC indexes and risk scores indicated that they were negatively correlated (R = -0.35, *P* = 6.5e-06); that is, the stem cell characteristics of GBM patients with low-risk scores were more significant (**Figure 11A**). It is generally believed that tumors with high TMB would respond better to immunotherapy and thus have a better prognosis. Based on mutation data of the TCGA GBM cohort, the differential analysis demonstrated that TMB was lower in the high-risk cluster than in the low-risk group (**Figure 11B**). Next, the result of the correlation analysis between TMB and risk score indicated that they were negatively correlated (R = -0.048, *p* = 0.049; **Figure 11C**). Interestingly, there was no significant correlation in the low-risk group (R = 0.044, *p* = 0.12; **Figure 11D**) and a negative correlation in the high-risk group (R = -0.012, *p* = 0.04; **Figure 11E**). To determine the specific distribution of somatic mutations, we constructed the waterfall diagrams of the two risk score groups, and the ten most frequently mutated genes in the high-risk cluster were PTEN, EGFR, TP53, TTN, NF1, MUC16, PIK3CA, LRP2, RYR2, and SPTA1 (**Figure 11F**), while, in the low-risk cluster were TP53, PTEN, TTN, EGFR, MUC16, ATRX, SPTA1, FLG, IDH1, and RYR2 (**Figure 11G**). The mutation frequencies of PTEN, EGFR, and NF1 in the low-risk cluster were lower than in the high-risk cluster, while the mutation frequencies of ATRX, IDH1, TP53, MUC16, and PIK3R1 in the low-risk cluster were higher than in the high-risk cluster (**Figures 11F, G**). Furthermore, common drugs or compounds were selected to detect the association between drug sensitivities and risk scores. Results of differential analyses of IC50 values between the two risk clusters indicated that IC50 values of bryostatin, midostaurin, mirdametinib, ponatinib, and tipifarnib in the high-risk cluster were lower than in the low-risk cluster. In comparison, the IC50 values of afatinib and elesclomol in the high-risk cluster were higher than in the low-risk cluster. Results suggested that drug sensitivity was somewhat associated with risk score (**Figures 11H–N**).

### Constructed a nomogram to predict survival

**Figure 12:**
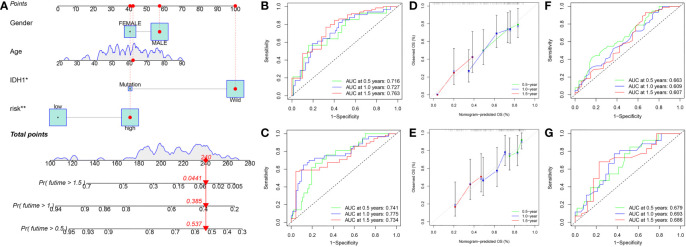
Constructed a nomogram in the training set. **(A)** Nomogram for predicting the 0.5-, 1.0-, and 1.5-year OS of GBM patients in the training set. **(B)** ROC curves based on the nomogram to predict OS at 0.5-, 1.0-, and 1.5-year in the training set. **(C)** ROC curves based on the nomogram to predict OS at 0.5-, 1.0-, and 1.5-year in the testing set. **(D)** Calibration curve of the nomogram to predict 0.5-, 1.0-, and 1.5-year OS in the training set. **(E)** Calibration curve of the nomogram to predict 0.5-, 1.0-, and 1.5-year OS in the testing set. **(F)** ROC curves based on IDH1 mutation status to predict OS at 0.5-, 1.0-, and 1.5-year in the training set. **(G)** ROC curves based on IDH1 mutation status to predict OS at 0.5-, 1.0-, and 1.5-year in the testing set. OS, overall survival; ROC, receiver operating characteristic.

To improve the applicability of this model in the clinic, a nomogram containing clinical parameters (age, sex, and IDH1 mutation status) and risk score was developed (**Figure 12A**). Based on the nomogram, the AUC values of 0.5-, 1.0-, and 1.5-year ROC curves for the prediction of OS were 0.716, 0.727, and 0.763 in the training set, respectively (**Figure 12B**), and were 0.741, 0.775, and 0.734 in the testing set, respectively (**Figure 12C**). Moreover, calibration curves showed that the predicted results were very close to the ideal results in the two sets (**Figures 12D, E**). Next, we detected the predictive performance of IDH1 mutation status alone. Based on IDH1 mutation status alone, the AUC values of 0.5-, 1.0-, and 1.5-year ROC curves for the prediction of OS were 0.663, 0.609, and 0.607 in the training set, respectively (**Figure 12F**), and were 0.679, 0.693, and 0.686 in the testing set, respectively (**Figure 12G**). Results indicated that the nomogram has excellent predictive performance and is better than IDH1 mutation status alone.

## Discussion

GBM is the most aggressive glioma, with a median survival of merely 15 months (13). Clinical observation found that the biological behavior and clinical prognosis of GBM are far from each other. Some GBM patients have a relatively long survival time, while others show a highly malignant outcome. WHO classification, the traditional histopathological diagnosis method, cannot accurately reflect the characteristics of GBM (14). It is of great clinical significance to explore novel prognostic markers and selectively administer different therapeutic strategies (15). With the popularization of genome sequencing, massive biological data provide more prognostic information, which complements the traditional WHO classification criteria. In recent years, according to the gene expression profiles obtained from the databases, many researchers have constructed prognostic models in GBM through various bioinformatic analysis methods. In particular, the expression profiles of several newly reported programmed cell death (PCD) types-related genes, including autophagy, pyroptosis, ferroptosis, and cuproptosis.

Autophagy, type II programmed cell death, is the process of phagocytosis of self-protein or organelles to form autophagic lysosomes and degradation of their contents, which meets the need for cell metabolism and the renewal of some organelles (16). Wang et al. developed a GBM risk model according to four autophagy-related genes, including STAM, MAPK8, LGALS8, and DIRAS3, and its 1-year AUC value was 0.644 (17). Pyroptosis, also known as inflammatory necrosis, is characterized by an intense inflammatory reaction caused by the rupture of cell membranes (18). Liang et al. constructed a risk score model in GBM patients according to four pyroptosis-related genes, including FOXP3, IRF3, CD274, and TP63, and its 1-year AUC value was 0.726 (19). Ferroptosis, iron-dependent programmed cell death, is featured lipid peroxidation of unsaturated fatty acids *via* Fe^2+^, ultimately leading to cell death (20). Xiao et al. constructed a prognostic model in GBM based on the expression profiles of five ferroptosis-related genes, including DUOX1, CDKN1A, GSS, ALOX5, and SQSTM1, and its 1-year AUC value was 0.680 (21). Similarly, Dong et al. constructed a risk model in GBM in light of five ferroptosis-related genes, including TFRC, STEAP3, NCOA4, AKR1C1, and AKR1C3, and its 3-year AUC value was 0.706 (22). With the gradual progress of research, currently, researchers focus on cuproptosis.

In March 2022, Tsvetkov et al. proposed for the first time a copper ion-dependent and novel programmed cell death type, namely cuproptosis. Research indicated that Cu^2+^ combines with the lipoylated components of the tricarboxylic acid cycle in the mitochondrial respiratory chain, resulting in the aggregation of lipoylated protein and down-regulation of iron-sulfur cluster protein, followed by proteotoxic stress as well as cell death (3). Moreover, research initially identified 12 CRGs, including CDKN2A, PDHB, GLS, LIPT1, FDX1, DLD, MTF1, ATP7B, LIAS, DLAT, PDHA1, and SLC31A1. Inspired by the above research, prognostic models based on the expression profiles of CRGs have been reported in many kinds of tumors. In renal carcinoma, Bian et al. established a risk model according to the expression profiles of three CRGs, including FDX1, DLAT, and CDKN2A, and its 1-year AUC value was 0.652 (7). In hepatocellular carcinoma, Zhang et al. constructed a cuproptosis-related prognostic model based on the expression profiles of four genes, including CAT, SLC27A5, EHHADH, and ALDH5A1, and its 1-year AUC value was 0.72 (23). In glioma, Wang et al. developed a cuproptosis-related-model in light of six genes, including H19, CHI3L1, CYTOR, IGFBP2, and C5orf38, and its 1-year AUC value was 0.898 (24). Similar research has been reported in low-grade glioma (25) and WHO 2/3 glioma (10). However, no cuproptosis-related prognostic model has been reported in GBM.

To establish a GBM prognostic model in light of CRGs, this study first performed a differentially expressed analysis of 12 CRGs between normal and GBM specimens and analyzed their prognostic significance. Based on the expression profiles of five prognostic CRGs, the cuproptosis subtypes (CRGcluster A and CRGcluster B) were identified on GBM samples. Second, we conducted a differentially expressed genes (DEGs) analysis of the two subtypes, followed by a prognosis analysis of the DEGs, and obtained 79 prognostic DEGs. According to the expression profiles of prognostic DEGs, gene subtypes were identified, and two subtypes, geneCluster A and geneCluster B were obtained. Then, through LASSO regression analysis in the training set, we established a risk score model containing five genes, and its AUC value of 1.0-year was 0.709. Validation was performed in the testing set, GSE83300 cohort, and GSE74187 cohort, and the AUC values of 1.0-year were 0.671, 0.731, and 0.731, respectively. Moreover, to improve the performance of our model, a nomogram was established, and its 1-year AUC values were 0.727 in the training set and 0.775 in the testing set. Compared with other PCD-related prognostic models, the prognostic prediction performance of our model may not be the most excellent. However, it is still superior to most reported PCD-related prognostic models of GBM, such as 0.644 for the autophagy-related model (17) and 0.680 (21) or 0.706 (22) for ferroptosis-related models.

The model constructed in this study included the following five genes: PDIA4, PILRB, DUSP6, PTPRN, and CBLN1. PDIA4, a member of the protein disulfide isomerase family, is mainly localized in the ER (26). Studies have reported that PDIA4 is related to mitochondrial apoptosis, aerobic glycolysis, and glucose metabolism (27, 28). In our research, PDIA4 is a high-risk gene. Consistently, Wang et al. reported that PDIA4 inhibits apoptosis and promotes the proliferation of glioblastoma *via* the PI3K/AKT/mTOR signaling pathway (29). DUSP6, dual-specificity phosphatase 6, is a member of a protein tyrosine phosphatases subfamily and modulates cell proliferation, differentiation, and apoptosis *via* the regulation of ERK signaling (30). In our risk score model, DUSP6 is a high-risk gene with a risk coefficient of 0.2448. Consistently, researchers have reported that the upregulation of DUSP6 plays a tumor-promoting role in GBM (31), and DUSP6 inhibition increases the radiosensitivity of GBM by regulating DNA damage repair (32). PTPRN, protein tyrosine phosphatase receptor type N, is located on band 2q35 and encodes a type I transmembrane protein. It mainly expresses in various endocrine cells and participates in neuroendocrine processes (33). In our study, PTPRN is a high-risk gene in GBM, whose risk coefficient is 0.3055. A study by Wang et al. consistently showed that PTPRN interacts with HSP90AA1 to activate PI3K/AKT signaling pathway and promote the proliferation and metastasis of high-grade gliomas (34). CBLN1, a member of the C1q family, is a synaptic organizer released by cerebellar granule cells and affects synapse formation and maintenance (35). Here, it’s a low-risk gene with a risk coefficient of -0.2095. In contrast, relatively few studies have been conducted on PILRB and CBLN1, and their relationship with tumors is poorly understood. The biological functions of most genes in this model are consistent with that reported previously, reflecting our results’ reliability.

TME has a significant effect on the development of tumors, and one of the most critical components of TME is tumor-infiltrating lymphocytes, including T cells and NK cells (36). Regulatory T cells are a unique T cells subset known as Foxp3^+^CD25^high^CD4^+^CD127^low^ Treg cells. Tregs weaken immune responses by inhibiting the proliferation of other T cells in TME (37). The poorer prognosis of GBM is always associated with higher Treg infiltration (38). Consistently, our research indicated that the risk score was positively correlated with the relative content of Tregs in GBM samples. As for NK cells, they are composed of cytotoxic effector lymphocytes and affect the anti-tumor innate immune response. NK cell infiltration into tumors is associated with better antitumor efficacy and patient prognosis (39). Consistently, results indicated that the risk score of GBM samples was negatively correlated with the content of activated NK cells while positively correlated with the content of resting NK cells. Macrophages are categorized into two main phenotypes. M1 macrophages are historically regarded as anti-tumor, while M2 macrophages contribute to many pro-tumorigenic outcomes in cancer through angiogenic regulation, immune suppression, tumor cell proliferation, and metastasis (40). However, in this study, M2 macrophages were lower in the high-risk score group than in the low-risk group. The effect of the tumor microenvironment on tumor biological behavior is the comprehensive effect of multiple immune cells, not a single immune cell. Although there were apparent differences in some kinds of immune cells between the two risk score groups, the immune score did not show a difference, and other regulatory mechanisms may be involved. Further studies on the effect of cuproptosis on the TME of GBM are needed to uncover the regulatory mechanisms of cuproptosis in GBM.

Activating immune checkpoints by malignant tumors can generate immunosuppressive microenvironments, and PD-1 and PD-L1 are the most studied immune checkpoints (41). Immune checkpoints, especially PD-1 and PD-L1, inhibit the T-cell activity or induce T-cell exhaustion and evade immune surveillance, which leads to a poor prognosis (42). Multiple clinical trials, including two phase III trials (NCT02667587 and NCT02617589), are ongoing to assess the potential of PD-1/PD-L1 checkpoint inhibitors, such as pembrolizumab and nivolumab, as monotherapy and combination therapy for glioblastoma (43). Consistent with previous published, in our study, the expression levels of many immune checkpoints in the high-risk group were higher than that in the low-risk group, including CD274 (PD-L1), PDCD1 (PD-1), CD276, NRP1, TNFSF14, TNFRSF18, etc.

In this research, there are two limitations. First, data were downloaded from public databases, and some clinical parameters, such as specific information on surgery and chemoradiotherapy, needed to be included. Second, all samples collected from our hospital were retrospective. In the future, prospective clinical research and experimental research will be required for further validation.

## Conclusion

In this research, by analyzing the expression profiles and clinical data of GBM downloaded from databases, a risk score model was constructed and verified based on CRGs. A nomogram was further developed to improve the applicability. We also studied the relationship between classification and TME and its guiding significance in immunotherapy or chemotherapy. This study provides a new idea for prognostic prediction and guidance of precise treatment of GBM.

## Data availability statement

The original contributions presented in the study are included in the article/**Supplementary Material**. Further inquiries can be directed to the corresponding authors.

## Ethics statement

The studies involving human participants were reviewed and approved by the ethics committee of Sun Yat-Sen Memorial Hospital. The patients/participants provided their written informed consent to participate in this study.

## Author contributions

MH and AL conceived the project. BZ and LX collected and analyzed the data. JL conducted experimental research. BZ wrote the manuscript, and MH revised it. All authors contributed to the article and approved the submitted version
