# Supplementary material for: Construction and validation of a cuproptosis-related prognostic model for glioblastoma
Source: Front Immunol. 2023 Feb 6;14:1082974. doi: 10.3389/fimmu.2023.1082974 (PMC9939522; doi:10.3389/fimmu.2023.1082974)
Supplement: Supplementary file 1 [file Table_1.docx]

| **Table S1: Clinical information of 209 glioblastoma multiforme patients** | | | | | | | |
| --- | --- | --- | --- | --- | --- | --- | --- |
| **Id** | **futime** | **fustat** | **Age** | **Gender** | **Grade** | **IDH1 mutation** | **Dataset** |
| TCGA_TCGA-76-4932 | 1458 | 1 | 50 | FEMALE | G4 | Wild | TCGA |
| TCGA_TCGA-76-4931 | 279 | 1 | 70 | FEMALE | G4 | Wild | TCGA |
| TCGA_TCGA-76-4929 | 111 | 1 | 76 | FEMALE | G4 | Wild | TCGA |
| TCGA_TCGA-76-4928 | 94 | 1 | 85 | FEMALE | G4 | Wild | TCGA |
| TCGA_TCGA-76-4927 | 535 | 1 | 58 | MALE | G4 | Wild | TCGA |
| TCGA_TCGA-76-4926 | 138 | 1 | 68 | MALE | G4 | Wild | TCGA |
| TCGA_TCGA-76-4925 | 146 | 1 | 76 | MALE | G4 | Wild | TCGA |
| TCGA_TCGA-41-5651 | 460 | 1 | 59 | FEMALE | G4 | Wild | TCGA |
| TCGA_TCGA-41-4097 | 6 | 1 | 63 | FEMALE | G4 | Wild | TCGA |
| TCGA_TCGA-41-3915 | 360 | 1 | 48 | MALE | G4 | Wild | TCGA |
| TCGA_TCGA-41-2572 | 406 | 1 | 67 | MALE | G4 | Wild | TCGA |
| TCGA_TCGA-41-2571 | 26 | 1 | 89 | MALE | G4 | Wild | TCGA |
| TCGA_TCGA-32-5222 | 585 | 1 | 66 | MALE | G4 | Wild | TCGA |
| TCGA_TCGA-32-4213 | 604 | 0 | 47 | FEMALE | G4 | Wild | TCGA |
| TCGA_TCGA-32-2638 | 766 | 1 | 67 | MALE | G4 | Wild | TCGA |
| TCGA_TCGA-32-2634 | 693 | 0 | 82 | MALE | G4 | Wild | TCGA |
| TCGA_TCGA-32-2632 | 269 | 1 | 80 | MALE | G4 | Wild | TCGA |
| TCGA_TCGA-32-2616 | 224 | 1 | 48 | FEMALE | G4 | Wild | TCGA |
| TCGA_TCGA-32-2615 | 485 | 1 | 62 | MALE | G4 | Wild | TCGA |
| TCGA_TCGA-32-1982 | 142 | 1 | 76 | FEMALE | G4 | Wild | TCGA |
| TCGA_TCGA-32-1980 | 36 | 1 | 72 | MALE | G4 | Wild | TCGA |
| TCGA_TCGA-32-1970 | 468 | 1 | 59 | MALE | G4 | Wild | TCGA |
| TCGA_TCGA-28-5220 | 388 | 1 | 67 | MALE | G4 | Wild | TCGA |
| TCGA_TCGA-28-5218 | 157 | 1 | 63 | MALE | G4 | Wild | TCGA |
| TCGA_TCGA-28-5216 | 415 | 0 | 52 | MALE | G4 | Wild | TCGA |
| TCGA_TCGA-28-5215 | 335 | 1 | 62 | FEMALE | G4 | Wild | TCGA |
| TCGA_TCGA-28-5213 | 298 | 0 | 72 | MALE | G4 | Wild | TCGA |
| TCGA_TCGA-28-5209 | 442 | 0 | 66 | FEMALE | G4 | Wild | TCGA |
| TCGA_TCGA-28-5208 | 544 | 1 | 52 | MALE | G4 | Wild | TCGA |
| TCGA_TCGA-28-5207 | 343 | 1 | 71 | MALE | G4 | unknow | TCGA |
| TCGA_TCGA-28-5204 | 454 | 1 | 72 | MALE | G4 | Wild | TCGA |
| TCGA_TCGA-28-2514 | 160 | 0 | 45 | MALE | G4 | Wild | TCGA |
| TCGA_TCGA-28-2513 | 222 | 0 | 69 | FEMALE | G4 | Wild | TCGA |
| TCGA_TCGA-28-2509 | 145 | 0 | 77 | FEMALE | G4 | Wild | TCGA |
| TCGA_TCGA-28-1753 | 37 | 0 | 53 | MALE | G4 | Wild | TCGA |
| TCGA_TCGA-28-1747 | 77 | 1 | 44 | MALE | G4 | Wild | TCGA |
| TCGA_TCGA-27-2528 | 480 | 1 | 62 | MALE | G4 | Wild | TCGA |
| TCGA_TCGA-27-2526 | 87 | 1 | 79 | FEMALE | G4 | Wild | TCGA |
| TCGA_TCGA-27-2524 | 231 | 1 | 56 | MALE | G4 | Wild | TCGA |
| TCGA_TCGA-27-2523 | 489 | 1 | 63 | MALE | G4 | Wild | TCGA |
| TCGA_TCGA-27-2521 | 510 | 1 | 34 | MALE | G4 | Mutation | TCGA |
| TCGA_TCGA-27-2519 | 550 | 1 | 48 | MALE | G4 | Wild | TCGA |
| TCGA_TCGA-27-1837 | 427 | 1 | 36 | MALE | G4 | Wild | TCGA |
| TCGA_TCGA-27-1835 | 648 | 1 | 53 | FEMALE | G4 | Wild | TCGA |
| TCGA_TCGA-27-1834 | 1233 | 1 | 56 | MALE | G4 | Wild | TCGA |
| TCGA_TCGA-27-1832 | 300 | 1 | 59 | FEMALE | G4 | Wild | TCGA |
| TCGA_TCGA-27-1831 | 505 | 1 | 66 | MALE | G4 | Wild | TCGA |
| TCGA_TCGA-27-1830 | 154 | 1 | 57 | MALE | G4 | Wild | TCGA |
| TCGA_TCGA-26-5139 | 48 | 0 | 65 | FEMALE | G4 | Wild | TCGA |
| TCGA_TCGA-26-5136 | 577 | 1 | 78 | FEMALE | G4 | Wild | TCGA |
| TCGA_TCGA-26-5135 | 270 | 1 | 72 | FEMALE | G4 | Wild | TCGA |
| TCGA_TCGA-26-5134 | 167 | 0 | 74 | MALE | G4 | Wild | TCGA |
| TCGA_TCGA-26-5133 | 452 | 0 | 59 | MALE | G4 | Wild | TCGA |
| TCGA_TCGA-26-5132 | 286 | 0 | 74 | MALE | G4 | unknow | TCGA |
| TCGA_TCGA-26-1442 | 953 | 0 | 43 | MALE | G4 | Mutation | TCGA |
| TCGA_TCGA-19-5960 | 455 | 1 | 56 | MALE | G4 | Wild | TCGA |
| TCGA_TCGA-19-4065 | 214 | 0 | 36 | MALE | G4 | Wild | TCGA |
| TCGA_TCGA-19-2629 | 737 | 1 | 60 | MALE | G4 | Mutation | TCGA |
| TCGA_TCGA-19-2625 | 124 | 1 | 76 | FEMALE | G4 | Wild | TCGA |
| TCGA_TCGA-19-2624 | 5 | 1 | 51 | MALE | G4 | Wild | TCGA |
| TCGA_TCGA-19-2620 | 148 | 1 | 70 | MALE | G4 | Wild | TCGA |
| TCGA_TCGA-19-2619 | 294 | 0 | 55 | FEMALE | G4 | unknow | TCGA |
| TCGA_TCGA-19-1787 | 385 | 1 | 48 | MALE | G4 | Wild | TCGA |
| TCGA_TCGA-19-1390 | 772 | 1 | 63 | FEMALE | G4 | Wild | TCGA |
| TCGA_TCGA-19-1389 | 141 | 1 | 51 | MALE | G4 | Wild | TCGA |
| TCGA_TCGA-19-0957 | 666 | 1 | 48 | FEMALE | G4 | Wild | TCGA |
| TCGA_TCGA-16-1045 | 883 | 1 | 49 | FEMALE | G4 | Wild | TCGA |
| TCGA_TCGA-16-0846 | 119 | 1 | 85 | MALE | G4 | Wild | TCGA |
| TCGA_TCGA-15-1444 | 1537 | 1 | 21 | MALE | G4 | unknow | TCGA |
| TCGA_TCGA-15-0742 | 419 | 1 | 65 | MALE | G4 | Wild | TCGA |
| TCGA_TCGA-14-2554 | 532 | 1 | 52 | FEMALE | G4 | Wild | TCGA |
| TCGA_TCGA-14-1829 | 218 | 0 | 57 | MALE | G4 | Wild | TCGA |
| TCGA_TCGA-14-1825 | 232 | 1 | 70 | MALE | G4 | Wild | TCGA |
| TCGA_TCGA-14-1823 | 543 | 1 | 58 | FEMALE | G4 | Wild | TCGA |
| TCGA_TCGA-14-1402 | 975 | 1 | 58 | FEMALE | G4 | unknow | TCGA |
| TCGA_TCGA-14-1034 | 485 | 1 | 60 | FEMALE | G4 | Wild | TCGA |
| TCGA_TCGA-14-0871 | 880 | 1 | 74 | FEMALE | G4 | Wild | TCGA |
| TCGA_TCGA-14-0817 | 164 | 1 | 69 | FEMALE | G4 | Wild | TCGA |
| TCGA_TCGA-14-0790 | 419 | 1 | 64 | FEMALE | G4 | Wild | TCGA |
| TCGA_TCGA-14-0789 | 342 | 1 | 54 | MALE | G4 | Wild | TCGA |
| TCGA_TCGA-14-0787 | 68 | 1 | 69 | MALE | G4 | Wild | TCGA |
| TCGA_TCGA-14-0781 | 29 | 1 | 49 | MALE | G4 | Wild | TCGA |
| TCGA_TCGA-14-0736 | 460 | 1 | 49 | MALE | G4 | Wild | TCGA |
| TCGA_TCGA-12-5299 | 98 | 1 | 56 | FEMALE | G4 | Wild | TCGA |
| TCGA_TCGA-12-5295 | 454 | 1 | 60 | FEMALE | G4 | Wild | TCGA |
| TCGA_TCGA-12-3653 | 442 | 1 | 34 | FEMALE | G4 | Wild | TCGA |
| TCGA_TCGA-12-3652 | 1062 | 1 | 60 | MALE | G4 | Wild | TCGA |
| TCGA_TCGA-12-3650 | 333 | 1 | 46 | MALE | G4 | Wild | TCGA |
| TCGA_TCGA-12-1597 | 675 | 1 | 62 | FEMALE | G4 | Wild | TCGA |
| TCGA_TCGA-12-0821 | 323 | 1 | 62 | MALE | G4 | Wild | TCGA |
| TCGA_TCGA-12-0619 | 1062 | 1 | 60 | MALE | G4 | Wild | TCGA |
| TCGA_TCGA-12-0618 | 395 | 1 | 49 | MALE | G4 | Wild | TCGA |
| TCGA_TCGA-12-0616 | 448 | 1 | 36 | FEMALE | G4 | Wild | TCGA |
| TCGA_TCGA-08-0386 | 548 | 1 | 74 | MALE | G4 | Wild | TCGA |
| TCGA_TCGA-06-5859 | 139 | 0 | 63 | MALE | G4 | Wild | TCGA |
| TCGA_TCGA-06-5858 | 187 | 0 | 45 | FEMALE | G4 | Wild | TCGA |
| TCGA_TCGA-06-5856 | 114 | 1 | 58 | MALE | G4 | Wild | TCGA |
| TCGA_TCGA-06-5418 | 83 | 1 | 75 | FEMALE | G4 | Wild | TCGA |
| TCGA_TCGA-06-5417 | 155 | 0 | 45 | FEMALE | G4 | Mutation | TCGA |
| TCGA_TCGA-06-5416 | 204 | 0 | 23 | FEMALE | G4 | Wild | TCGA |
| TCGA_TCGA-06-5414 | 273 | 0 | 61 | MALE | G4 | Wild | TCGA |
| TCGA_TCGA-06-5413 | 268 | 0 | 67 | MALE | G4 | Wild | TCGA |
| TCGA_TCGA-06-5412 | 138 | 1 | 78 | FEMALE | G4 | Wild | TCGA |
| TCGA_TCGA-06-5411 | 254 | 1 | 51 | MALE | G4 | Wild | TCGA |
| TCGA_TCGA-06-5410 | 108 | 1 | 72 | FEMALE | G4 | Wild | TCGA |
| TCGA_TCGA-06-5408 | 357 | 1 | 54 | FEMALE | G4 | Wild | TCGA |
| TCGA_TCGA-06-2570 | 958 | 0 | 21 | FEMALE | G4 | Mutation | TCGA |
| TCGA_TCGA-06-2569 | 13 | 0 | 24 | FEMALE | G4 | Wild | TCGA |
| TCGA_TCGA-06-2567 | 133 | 1 | 65 | MALE | G4 | Wild | TCGA |
| TCGA_TCGA-06-2565 | 506 | 1 | 59 | MALE | G4 | Wild | TCGA |
| TCGA_TCGA-06-2564 | 181 | 0 | 50 | MALE | G4 | Wild | TCGA |
| TCGA_TCGA-06-2563 | 932 | 0 | 72 | FEMALE | G4 | Wild | TCGA |
| TCGA_TCGA-06-2562 | 382 | 1 | 81 | MALE | G4 | Wild | TCGA |
| TCGA_TCGA-06-2561 | 537 | 1 | 53 | FEMALE | G4 | Wild | TCGA |
| TCGA_TCGA-06-2559 | 150 | 1 | 83 | MALE | G4 | Wild | TCGA |
| TCGA_TCGA-06-2558 | 380 | 1 | 75 | FEMALE | G4 | Wild | TCGA |
| TCGA_TCGA-06-2557 | 33 | 1 | 76 | MALE | G4 | Wild | TCGA |
| TCGA_TCGA-06-1804 | 414 | 1 | 81 | FEMALE | G4 | Wild | TCGA |
| TCGA_TCGA-06-0882 | 632 | 1 | 30 | MALE | G4 | Wild | TCGA |
| TCGA_TCGA-06-0878 | 218 | 0 | 74 | MALE | G4 | Wild | TCGA |
| TCGA_TCGA-06-0750 | 28 | 1 | 43 | MALE | G4 | Wild | TCGA |
| TCGA_TCGA-06-0749 | 82 | 1 | 50 | MALE | G4 | Wild | TCGA |
| TCGA_TCGA-06-0747 | 82 | 1 | 53 | MALE | G4 | Wild | TCGA |
| TCGA_TCGA-06-0745 | 239 | 1 | 59 | MALE | G4 | Wild | TCGA |
| TCGA_TCGA-06-0744 | 1426 | 1 | 66 | MALE | G4 | Wild | TCGA |
| TCGA_TCGA-06-0743 | 803 | 1 | 69 | MALE | G4 | Wild | TCGA |
| TCGA_TCGA-06-0686 | 432 | 1 | 53 | MALE | G4 | Wild | TCGA |
| TCGA_TCGA-06-0649 | 64 | 1 | 73 | FEMALE | G4 | Wild | TCGA |
| TCGA_TCGA-06-0646 | 175 | 1 | 60 | MALE | G4 | Wild | TCGA |
| TCGA_TCGA-06-0645 | 175 | 1 | 55 | FEMALE | G4 | Wild | TCGA |
| TCGA_TCGA-06-0644 | 384 | 1 | 71 | MALE | G4 | Wild | TCGA |
| TCGA_TCGA-06-0238 | 405 | 1 | 46 | MALE | G4 | Wild | TCGA |
| TCGA_TCGA-06-0221 | 603 | 1 | 31 | MALE | G4 | Mutation | TCGA |
| TCGA_TCGA-06-0219 | 22 | 1 | 67 | MALE | G4 | Wild | TCGA |
| TCGA_TCGA-06-0211 | 360 | 1 | 47 | MALE | G4 | Wild | TCGA |
| TCGA_TCGA-06-0210 | 225 | 1 | 72 | FEMALE | G4 | Wild | TCGA |
| TCGA_TCGA-06-0190 | 317 | 1 | 62 | MALE | G4 | Wild | TCGA |
| TCGA_TCGA-06-0187 | 828 | 1 | 69 | MALE | G4 | Wild | TCGA |
| TCGA_TCGA-06-0184 | 2126 | 1 | 63 | MALE | G4 | Wild | TCGA |
| TCGA_TCGA-06-0178 | 2681 | 1 | 38 | MALE | G4 | unknow | TCGA |
| TCGA_TCGA-06-0174 | 98 | 1 | 54 | MALE | G4 | Wild | TCGA |
| TCGA_TCGA-06-0171 | 399 | 1 | 65 | MALE | G4 | Wild | TCGA |
| TCGA_TCGA-06-0168 | 598 | 1 | 59 | FEMALE | G4 | Wild | TCGA |
| TCGA_TCGA-06-0158 | 329 | 1 | 73 | MALE | G4 | Wild | TCGA |
| TCGA_TCGA-06-0157 | 97 | 1 | 63 | FEMALE | G4 | Wild | TCGA |
| TCGA_TCGA-06-0156 | 178 | 1 | 57 | MALE | G4 | unknow | TCGA |
| TCGA_TCGA-06-0152 | 375 | 1 | 68 | MALE | G4 | Wild | TCGA |
| TCGA_TCGA-06-0141 | 313 | 1 | 62 | MALE | G4 | Wild | TCGA |
| TCGA_TCGA-06-0139 | 362 | 1 | 40 | MALE | G4 | Wild | TCGA |
| TCGA_TCGA-06-0138 | 737 | 1 | 43 | MALE | G4 | unknow | TCGA |
| TCGA_TCGA-06-0132 | 771 | 1 | 49 | MALE | G4 | Wild | TCGA |
| TCGA_TCGA-06-0130 | 394 | 1 | 54 | MALE | G4 | Wild | TCGA |
| TCGA_TCGA-06-0129 | 1024 | 1 | 30 | MALE | G4 | Mutation | TCGA |
| TCGA_TCGA-06-0125 | 1448 | 1 | 63 | FEMALE | G4 | Wild | TCGA |
| TCGA_TCGA-02-2486 | 618 | 1 | 64 | MALE | G4 | Wild | TCGA |
| TCGA_TCGA-02-2485 | 470 | 0 | 53 | MALE | G4 | Wild | TCGA |
| TCGA_TCGA-02-2483 | 466 | 0 | 43 | MALE | G4 | Mutation | TCGA |
| TCGA_TCGA-02-0055 | 76 | 1 | 62 | FEMALE | G4 | Wild | TCGA |
| TCGA_TCGA-02-0047 | 448 | 1 | 78 | MALE | G4 | Wild | TCGA |
| GSE83300_GSM2198655 | 283.2 | 1 | 37 | MALE | G4 | unknow | GSE83300 |
| GSE83300_GSM2198654 | 380.7 | 1 | 55 | MALE | G4 | unknow | GSE83300 |
| GSE83300_GSM2198653 | 333.3 | 1 | 52 | MALE | G4 | unknow | GSE83300 |
| GSE83300_GSM2198652 | 952.8 | 1 | 38 | MALE | G4 | unknow | GSE83300 |
| GSE83300_GSM2198651 | 643.2 | 0 | 28 | MALE | G4 | unknow | GSE83300 |
| GSE83300_GSM2198650 | 295.8 | 1 | 68 | FEMALE | G4 | unknow | GSE83300 |
| GSE83300_GSM2198649 | 582.9 | 1 | 43 | FEMALE | G4 | unknow | GSE83300 |
| GSE83300_GSM2198648 | 331.5 | 1 | 52 | MALE | G4 | unknow | GSE83300 |
| GSE83300_GSM2198647 | 185.4 | 1 | 40 | MALE | G4 | unknow | GSE83300 |
| GSE83300_GSM2198646 | 684.6 | 0 | 54 | FEMALE | G4 | unknow | GSE83300 |
| GSE83300_GSM2198645 | 585 | 1 | 47 | MALE | G4 | unknow | GSE83300 |
| GSE83300_GSM2198644 | 541.5 | 1 | 27 | FEMALE | G4 | unknow | GSE83300 |
| GSE83300_GSM2198643 | 357.9 | 1 | 57 | FEMALE | G4 | unknow | GSE83300 |
| GSE83300_GSM2198642 | 557.4 | 1 | 33 | FEMALE | G4 | unknow | GSE83300 |
| GSE83300_GSM2198641 | 555.3 | 1 | 40 | FEMALE | G4 | unknow | GSE83300 |
| GSE83300_GSM2198640 | 276.3 | 0 | 43 | FEMALE | G4 | unknow | GSE83300 |
| GSE83300_GSM2198639 | 1273.2 | 0 | 18 | MALE | G4 | unknow | GSE83300 |
| GSE83300_GSM2198638 | 455.7 | 1 | 57 | FEMALE | G4 | unknow | GSE83300 |
| GSE83300_GSM2198637 | 277.2 | 1 | 45 | FEMALE | G4 | unknow | GSE83300 |
| GSE83300_GSM2198636 | 473.4 | 0 | 56 | FEMALE | G4 | unknow | GSE83300 |
| GSE83300_GSM2198635 | 522.6 | 1 | 60 | FEMALE | G4 | unknow | GSE83300 |
| GSE83300_GSM2198634 | 525.6 | 1 | 59 | MALE | G4 | unknow | GSE83300 |
| GSE83300_GSM2198633 | 576 | 1 | 35 | FEMALE | G4 | unknow | GSE83300 |
| GSE83300_GSM2198632 | 779.1 | 0 | 43 | unknow | G4 | unknow | GSE83300 |
| GSE83300_GSM2198631 | 413.4 | 1 | 42 | MALE | G4 | unknow | GSE83300 |
| GSE83300_GSM2198630 | 1074 | 1 | 38 | FEMALE | G4 | unknow | GSE83300 |
| GSE83300_GSM2198629 | 390.6 | 1 | 58 | FEMALE | G4 | unknow | GSE83300 |
| GSE83300_GSM2198628 | 239.7 | 1 | 37 | MALE | G4 | unknow | GSE83300 |
| GSE83300_GSM2198627 | 659.7 | 0 | 24 | FEMALE | G4 | unknow | GSE83300 |
| GSE83300_GSM2198626 | 814.8 | 1 | 37 | MALE | G4 | unknow | GSE83300 |
| GSE83300_GSM2198625 | 351 | 1 | 38 | MALE | G4 | unknow | GSE83300 |
| GSE83300_GSM2198624 | 1010.1 | 1 | 44 | FEMALE | G4 | unknow | GSE83300 |
| GSE83300_GSM2198623 | 320.4 | 1 | 62 | MALE | G4 | unknow | GSE83300 |
| GSE83300_GSM2198622 | 195.3 | 1 | 56 | MALE | G4 | unknow | GSE83300 |
| GSE83300_GSM2198621 | 397.5 | 1 | 39 | FEMALE | G4 | unknow | GSE83300 |
| GSE83300_GSM2198620 | 490.2 | 1 | 59 | MALE | G4 | unknow | GSE83300 |
| GSE83300_GSM2198619 | 1047.6 | 0 | 45 | FEMALE | G4 | unknow | GSE83300 |
| GSE83300_GSM2198618 | 377.7 | 1 | 55 | MALE | G4 | unknow | GSE83300 |
| GSE83300_GSM2198617 | 1286.1 | 0 | 28 | FEMALE | G4 | unknow | GSE83300 |
| GSE83300_GSM2198616 | 546.3 | 1 | 56 | MALE | G4 | unknow | GSE83300 |
| GSE83300_GSM2198615 | 499.2 | 1 | 42 | FEMALE | G4 | unknow | GSE83300 |
| GSE83300_GSM2198614 | 134.1 | 1 | 42 | FEMALE | G4 | unknow | GSE83300 |
| GSE83300_GSM2198613 | 948.9 | 0 | 61 | MALE | G4 | unknow | GSE83300 |
| GSE83300_GSM2198612 | 1387.8 | 1 | 32 | MALE | G4 | unknow | GSE83300 |
| GSE83300_GSM2198611 | 766.5 | 1 | 54 | MALE | G4 | unknow | GSE83300 |
| GSE83300_GSM2198610 | 407.4 | 1 | 35 | FEMALE | G4 | unknow | GSE83300 |
| GSE83300_GSM2198609 | 249.6 | 1 | 64 | MALE | G4 | unknow | GSE83300 |
| GSE83300_GSM2198608 | 375.9 | 1 | 46 | MALE | G4 | unknow | GSE83300 |
| GSE83300_GSM2198607 | 1281.3 | 0 | 35 | FEMALE | G4 | unknow | GSE83300 |
| GSE83300_GSM2198606 | 518.7 | 1 | 49 | FEMALE | G4 | unknow | GSE83300 |
