# Supplementary material for: Construction and validation of a cuproptosis-related prognostic model for glioblastoma
Source: Front Immunol. 2023 Feb 6;14:1082974. doi: 10.3389/fimmu.2023.1082974 (PMC9939522; doi:10.3389/fimmu.2023.1082974)
Supplement: Supplementary file 2 [file Table_2.docx]

| Table S2: The primer sequences for qRT-PCR. | |
| --- | --- |
| **Gene** | **Primer Sequence** |
| PDIA4 | F: 5'-GCGCTTAACCGGCGCGTACCT-3' |
|  | R: 5'-TTCCGGAAAGCTTCGAGCTAC-3' |
| DUSP6 | F: 5'-GGCCTTGGACTGACTGGTCA-3' |
|  | R: 5'-CGCGATATTGCAATGCCTGC-3' |
| PTPRN | F: 5'-GGCCTTTGCAAACATGCACAT-3' |
|  | R: 5'-TAAATCCAAGCGCGGCCATGC-3' |
| PILRB | F: 5'-AACCTATAGGCGACGTGCA-3' |
|  | R: 5'-GCGCGATTAACCGACTCCG-3' |
| CBLN1 | F: 5'-ATTAGCCGGTTAACTGACGG-3' |
|  | R: 5'-GGCCAATTACACGTGTCAGT-3' |
| GAPDH | F: 5'-AATGCGCGATATGCATTTAACCT-3' |
|  | R: 5'-TTGGCCAACGCGATATGCGCATG-3' |
