# Supplementary material for: Construction and validation of a cuproptosis-related prognostic model for glioblastoma
Source: Front Immunol. 2023 Feb 6;14:1082974. doi: 10.3389/fimmu.2023.1082974 (PMC9939522; doi:10.3389/fimmu.2023.1082974)
Supplement: Supplementary file 3 [file Table_3.docx]

| Table S3. Univariate Cox regression analysis of 5 CRGs associated with OS in GBM patients. | | | |
| --- | --- | --- | --- |
| id | **HR** | **95.0% CI** | **p-value** |
| ATP7B | 0.700997246 | 0.573-0.858 | 5.40E-04 |
| SLC31A1 | 1.874992484 | 1.398-2.515 | 3.30E-05 |
| DLD | 2.173501332 | 1.384-3.412 | 8.70E-04 |
| MTF1 | 1.930261804 | 1.101-3.384 | 0.021 |
| CDKN2A | 1.414072628 | 1.133-1.771 | 0.003 |
